# Chinese sacbrood virus mediates m6A modification to target and suppress the expression of hemolymph maintenance gene AF9, exacerbating bee infections

**DOI:** 10.1128/jvi.02117-24

**Published:** 2025-02-03

**Authors:** Hua Bai, Yueyu Ma, Huitong Qiu, Yang Qi, Yingshuo Huang, Yaxi Guo, Li Sun, Ming Li, Dongliang Fei, Mingxiao Ma, Yuming Liu

**Affiliations:** 1 College Animal Husbandry and Veterinary, Jinzhou Medical University 154516 , Jinzhou, Liaoning, China; Wageningen University & Research, Wageningen, Netherlands

**Keywords:** CSBV, AF9, m6A modification, immune suppression, broad-spectrum antiviral

## Abstract

The Chinese sacbrood virus (CSBV) severely threatens the beekeeping industry, wherein 3- to 5-day-old larvae in the critical differentiation stage are highly susceptible to low levels of CSBV exposure. Once infected, larvae cannot undergo normal pupation, but the pathogenic mechanism remains unclear. Previous studies have shown that m6A modification plays an important regulatory role in larval development during the critical differentiation stage. However, it is unknown whether CSBV infection affects the pupation of honeybee larvae by altering m6A modification. Here, a novel immunoregulatory factor, AF9, was identified in honeybee larvae through combined methylated RNA immunoprecipitation sequencing (MeRIP-seq) and RNA-seq analysis following CSBV infection. Enzyme-linked immunosorbent assay (ELISA) quantification of m6A methylation in total RNA and MeRIP-qPCR further revealed that CSBV infection of honeybee larvae inhibits the expression of *AF9* via m6A modification, thereby hindering the host innate immune response and promoting CSBV replication. MeRIP-qPCR was then used to demonstrate that AcMETTL3 targets and modifies *AF9* mRNA, thereby inhibiting *AF9* expression. Homology and functional analysis of human-derived *AF9* (MLLT3) suggested that AF9 exerted a similar effect as MLLT3 on honeybee hemolymph functioning. dsRNA was then fed to silence genes, followed by RNA extraction and expression analysis from hemolymph. Downregulation of *AF9* expression led to decreased numbers of live cells in the hemolymph of honeybee larvae and a reduction in phenoloxidase activity, thereby inhibiting the host immune response. Finally, an *Apis mellifera* pupation infection model was constructed to further explore the antiviral activities associated with *AmAF9. AmAF9* exerted a similarly significant antiviral effect against deformed wing virus (DWV) and acute bee paralysis virus (ABPV) infections in *Apis mellifera* pupae. These results indicate that CSBV infection promotes overall m6A modification in the host and inhibits the expression of *AF9* through AcMETTL3 targeting, leading to host immunosuppression and exacerbating honeybee infection. Similarly, *AF9* is stably expressed in *Apis mellifera* and exhibits the same antiviral effect, making it a broad-spectrum target in honeybee viruses.

**IMPORTANCE:**

The Chinese sacbrood virus (CSBV) poses a serious threat to the health of *Apis cerana* colonies, yet its specific pathogenic mechanism remains unclear. This study shows that infection with CSBV can enhance overall m6A modification levels in *Apis cerana* larvae and suppress the expression of *AF9* by promoting targeting of AcMETTL3, thereby inhibiting the innate immune response and exacerbating CSBV infection. Further analyses indicated that *AF9* functions similarly as the mammalian homologous gene *MLLT3* by maintaining normal functions of hemolymph. Moreover, *AF9* can also significantly inhibit infections by common *Apis mellifera* viruses. In summary, a new mechanism is detailed here by which CSBV escapes the host’s innate immune response by enhancing m6A modification to target and suppress the immune response gene *AF9*. This study also provides new insights into the mechanisms by which bee viruses inhibit host immune responses and suggests that *AF9* may serve as a potential new broad-spectrum antiviral target in bees.

## INTRODUCTION

*Apis cerana* is one of the important bee species among pollinators of crops and wild plants that is facing a serious threat from infection by the Sacbrood virus (SBV) (1). The Chinese sacbrood virus (CSBV) evolved from other SBVs in a geographic-dependent manner and poses a significant threat to the beekeeping industry due to substantial economic losses (2). CSBV is a small RNA virus belonging to the iflavirus, is non-enveloped, and exhibits a genetic composition similar to SBVs that infect European honeybees. The virus comprises a single-stranded positive-sense RNA molecule with a genome size of 8.8 kbp that encodes a large open reading frame (ORF). The structural genes are located at the 5′ end, but the encoded structural proteins are encoded elsewhere, with CSBV encoding four structural proteins (VP1, VP2, VP3, and VP4), and the non-structural genes located at the 3′ end (3). CSBV primarily spreads horizontally through feeding. Once the virus infects a bee colony, it causes larvae to form liquid sacs and fail to normally pupate. Although adult bees do not show clinical symptoms, their lifespans can be shortened, while their foraging and flying abilities may decline, and they may escape from the colony, ultimately leading to the collapse of the entire colony (1, 4). CSBV has crossed species barriers, because it naturally infects *Apis mellifera* and causes mass larval mortality (5). The 3- to 5-day-old larval stage is a critical differentiation period for honeybee larvae development and the peak period for susceptibility to CSBV infection. Notably, the m6A post-transcriptional modification of RNA plays an important regulatory developmental role in larvae during this period (6, 7). Consequently, interactions between m6A modification and CSBV-infected larvae warrant investigation.

The m6A modification is the most common post-transcriptional modification of RNA in eukaryotes and plays an important role in physiological and pathological processes via its mediation by specific RNA-modifying enzymes that achieve reversible regulation through RNA methyltransferases and demethylases (8). m6A modification of human circular RNA can inhibit innate immune responses (9). Furthermore, m6A modification also plays a significant role in behavioral performance and sex determination of fruit flies. Specifically, knockdown of the m6A methyltransferase Dm ime4 in the encapsulated cells of fruit flies results in decreased Chic protein levels that directly affect somatic permeability barriers, leading to germ cell death and reduced fertility in male fruit flies (10). Additionally, m6A methylation of the silkworm genome (*Bombyx mori*) is negatively correlated with active gene transcription, suggesting a key role of m6A modification in controlling their cell cycles (11). The roles of m6A modification in viral infections have gradually garnered interest in recent years. m6A modification has a negative regulatory effect on the RNA replication of Zika virus (ZIKV) and hepatitis C virus (HCV), albeit via different negative regulatory mechanisms. The negative regulatory modification of m6A by ZIKV inhibits replication by promoting viral transcript degradation, while HCV inhibits the production of infectious viral particles by competing with viral RNA for nucleocapsid proteins through YTHDF proteins, thereby suppressing viral replication (12). Similarly, a regulatory relationship exists between m6A modification and insect viruses, wherein m6A modification directly regulates the expression of the structural protein VP39 of *Bombyx mori* nucleopolyhedrovirus (BmNPV) (13). The above observations indicate that m6A modification may be a novel epigenetic mechanism that regulates insect virus infections, thereby providing a new disease-resistant strategy for preventing insect diseases.

Unlike mammals, bees have relatively simple immune systems that possess innate immunity and lack complete acquired immunity (14, 15). Innate immunity comprises humoral immunity and cellular immunity (primarily mediated by hemolymph). Antiviral defense mechanisms in bee humoral immunity include RNA interference (RNAi), autophagy, and conserved immune pathways, including the Jak/STAT (Janus kinase/signal transducer and activator of transcription), JNK (c-Jun N-terminal kinase), MAPK (mitogen-activated protein kinase), and NF-κB-mediated TOLL and IMD (immune deficiency) pathways (4, 16, 17). In addition, cellular immunity mediated by hemolymph clears foreign-invading pathogens through phagocytosis, melanization, encapsulation, and the phenoloxidase system (18, 19). The activation of phenoloxidase generates reactive oxygen species (e.g., O^-^, O_3_, and H_2_O_2_), further enhancing cellular immune responses and cascading the activation of humoral immune responses (4, 16, 20). The growth and development of bees require a complete metamorphosis process, during which the body cavity of bee larvae is primarily filled with hemolymph, fat bodies, and underdeveloped intestines. Consequently, the defensive role of hemolymph is particularly important in protecting bee larvae from external pathogen invasion. Thus, exploring and identifying host genes (antiviral targets) that can enhance or maintain the normal composition and function of hemolymph are critical for evaluating the capacity to control bee pathogens.

In this study, a novel gene, *AF9*, was identified, which plays a significant regulatory role in the innate immunity of bees. Moreover, a novel immunosuppressive mechanism was identified following CSBV infection in bee larvae, wherein CSBV infection enhances the overall levels of m6A modification and mediates the completion of the m6A modification of *AF9* mRNA via the methylation modification protein METTL3, thereby inhibiting the expression of *AF9*. In addition to positively regulating multiple immune pathways involved in bee humoral immunity (i.e., via the RNAi, Jak/STAT, TOLL, and IMD pathways, among others), *AF9* also plays an important maintenance role in the primary components and functions of bee larval hemolymph (i.e., cellular immunity). *AF9*-suppressed bee larvae exhibited higher levels of viral replication, pathological changes, and mortality after CSBV infection. Furthermore, the broad-spectrum antiviral properties of *AF9* against various bee viruses were validated in an established *Apis mellifera* pupae infection model. These results suggest that *AF9* is a potentially important antiviral target and provide new insights into immunosuppressive mechanisms triggered by bee virus infections in hosts.

## RESULTS

### Optimization of the CSBV infection model in *Apis cerana* larvae

**Fig 1:**
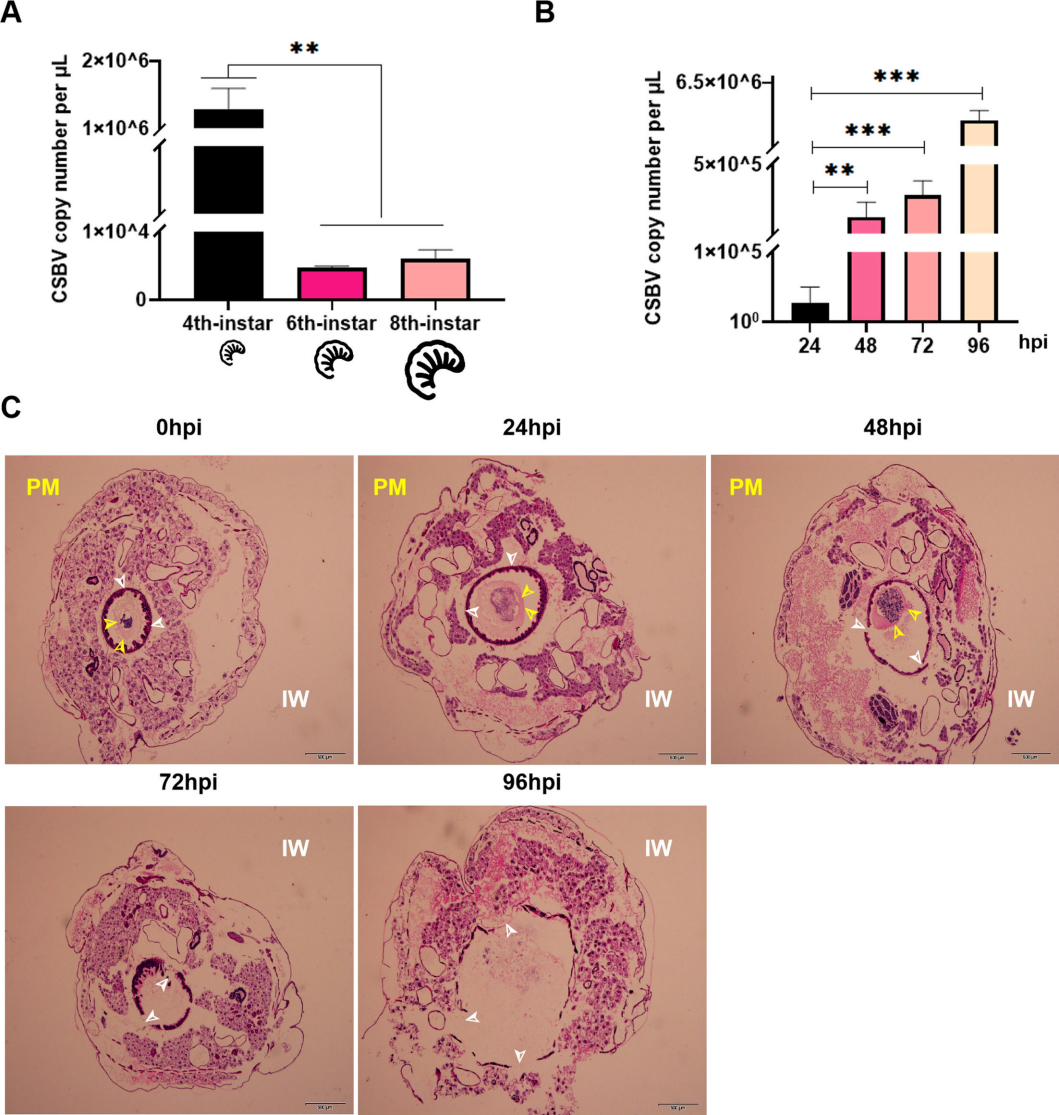
Optimization of the CSBV infection model in honey bee larvae. CSBV copy numbers were detected in honeybee larvae of different ages (4, 6, and 8 days old) and at a uniform pupation age (10 days old) (**A**). CSBV copy numbers were detected at different time points (24, 48, 72, and 96 hpi) in 4-day-old honeybee larvae (**B**) along with pathological changes (e.g., disappearance of the PM, intestinal dilation, and IW rupture) in larval bodies (**C**). Statistical analysis of grouped comparisons was evaluated by Student’s *t*-test (*P < 0.05, **P < 0.01, ***P < 0.001). Data shown are means ± SD, *n* = 5.

To optimize the standardized infection model of CSBV, the age of artificially reared larvae susceptible to CSBV and the optimal times for viral infection were evaluated. First, 4-, 6-, and 8-day-old *Apis cerana* larvae were fed equal doses of CSBV (1 × 10^7^ copies/μL). Considering that CSBV characteristically hinders normal larval pupation, samples were uniformly collected at the pupation age of honeybee larvae (10 days old) to detect CSBV copies. The 4-day-old (youngest aged) larvae were the most sensitive to CSBV infection, while the viral copy numbers in 6- or 8-day-old larvae after infection until pupation were lower and not significantly different (Fig. 1A). Four-day-old larvae were consequently used as infection targets and fed equal amounts of CSBV, followed by sampling at 24, 48, 72, and 96 hours postinfection (hpi) to detect viral copy levels. Larvae exhibited significant levels of viral copies and pathological changes (e.g., disappearance of the peritophagous membrane [PM], intestinal dilation, and intestinal wall [IW] rupture) at 48 hpi, and the viral copies and degree of pathological changes in intestines gradually increased as infections progressed (Fig. 1B and C). Thus, 4-day-old larvae were the optimal infection target, while 48 hours of infection was the peak infection time.

### Screening host genes that regulate CSBV replication with combined MeRIP-seq and RNA-seq

**Fig 2:**
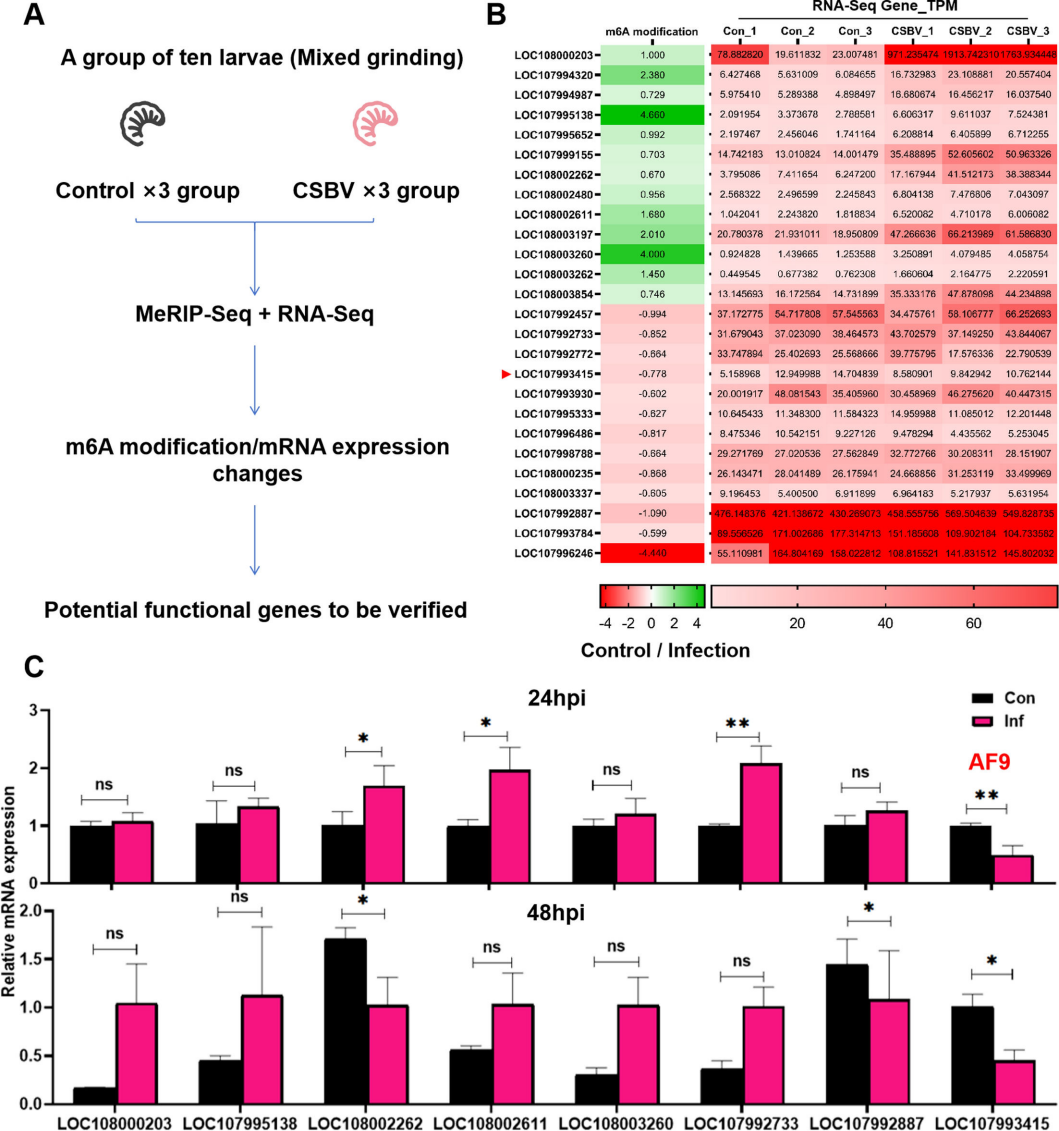
Identifying potential antiviral genes by combining MeRIP-seq and RNA-seq. (**A**) Sequencing workflow for MeRIP-seq and RNA-seq of samples from two groups (CSBV infected and non-infected) of 4-day-old larvae after 48 hours of treatment. (**B**) Relative expression levels of the 26 genes most differentially expressed in control and infection groups. (**C**) The expression levels of eight genes selected from those in (**B**) and analyzed at 24 and 48 hours post-CSBV infection. Statistical analysis of grouped comparisons was evaluated with Student’s *t*-tests (*P < 0.05, **P < 0.01, ***P < 0.001). Data shown are means ± SD, *n* = 5.

Combined methylated RNA immunoprecipitation sequencing (MeRIP-seq) and RNA-seq analyses were used to investigate the relationships between m6A modification of host genes and mRNA expression levels at 48 hpi after infection by CSBV in 4-day-old larvae. Treatment groups included control and infected groups that were each divided into three replicates, with 10 larvae in each replicate. The 10 larvae in each replicate were pooled for sequencing and comparison to the control group (Fig. 2A). The combined analysis indicated that CSBV infection can induce changes in host m6A modification, wherein the overall mRNA methylation level was negatively correlated with mRNA expression levels (we utilized publicly available RNA-seq and MeRIP-seq data, accession number PRJNA1068023). In this data set, the m6A abundance of 61 genes was negatively correlated with gene expression (Fig. S2). We first randomly divided the 61 target genes into three groups for functional validation to improve the efficiency of the selected target genes. The 26 genes (the first group) in this data set that exhibited a high correlation between methylation modification and mRNA expression differences were selected for additional analysis (Fig. 2B; Tables S3 and S4). We further validated eight of these genes using qPCR, and the other 18 genes will be tested in subsequent studies. Validation results demonstrated that *AF9* was significantly downregulated at both 24 and 48 hpi, consistent with the transcriptomics analyses, while the differences relative to the controls in other genes were not significant (Fig. 2C). *AF9* was consequently further investigated to understand its role in regulating viral replication.

### *AF9* gene analysis and comparison to human-derived *AF9* (MLLT3)

**Fig 3:**
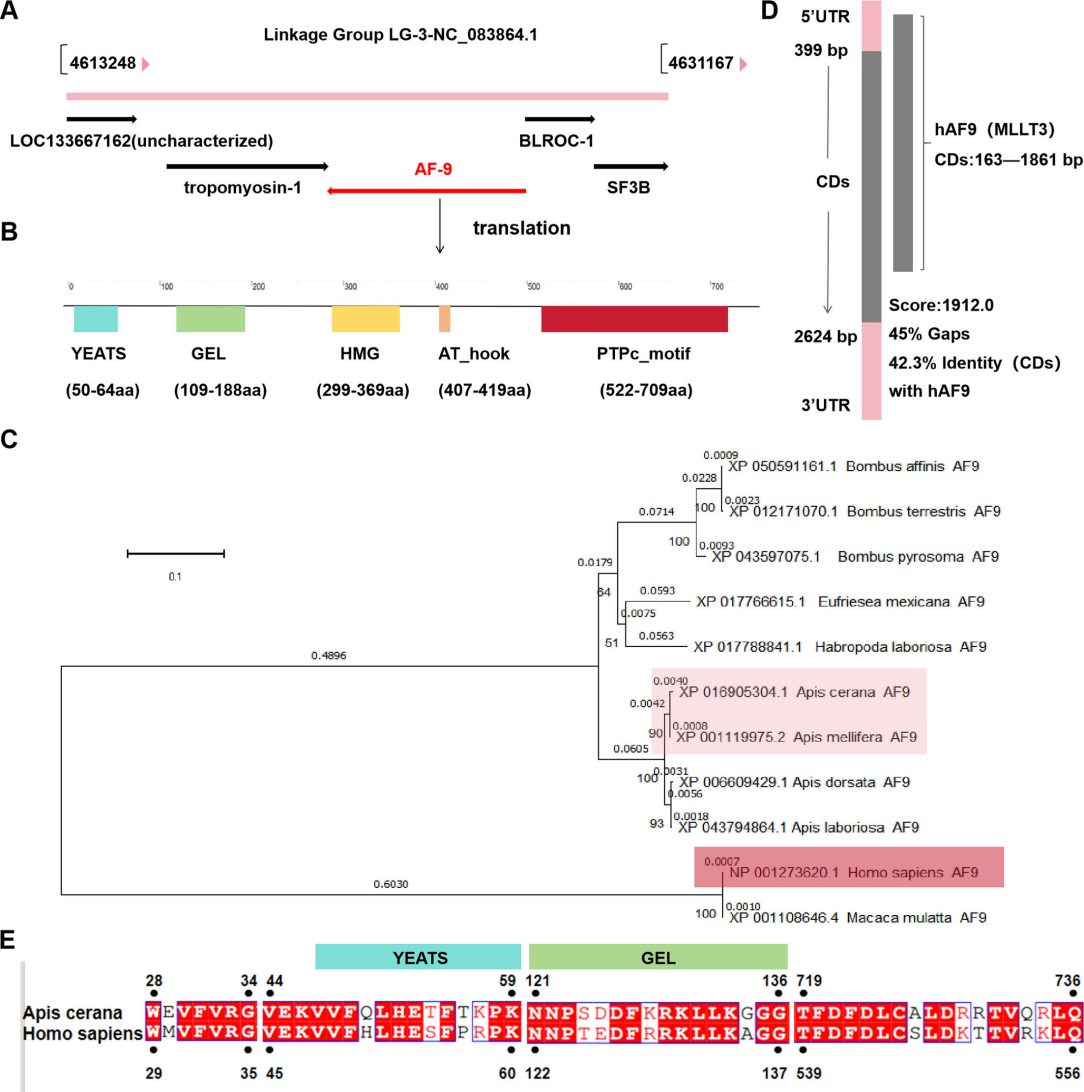
AcAF9 whole genome information and homology analysis with human-derived AF9 (MLLT3). (**A**) Genomic location of AcAF9 (red) and the relationship with gene NC_083864.1 (pink). (**B**) The amino acid functional domains of AcAF9 were predicted and are shown. (**C**) Phylogenetic analysis of the AF9 amino acid sequences from *Apis cerana* and other species. (**D and E**) Genetic sequence and amino acid alignment comparison between AcAF9 and MLLT3.

The location of *AF9* in the genome was determined to be transcribed from the gene locus NC_083864.1 of the *Apis cerana* that is situated between the genes *BLROC-1* and *tropomyosin-1* (Fig. 3A). The Simple Modular Architecture Research Tool (SMART) tool was then used to predict the amino acid functional domains of AcAF9 (Fig. 3B) and conduct phylogenetic analysis. These analyses indicated that *Apis mellifera* AF9 (AmAF9) and AcAF9 are genetically, evolutionarily correlated with human AF9 (MLLT3), and AmAF9 has a shorter evolutionary distance compared to AcAF9 (Fig. 3C). To further investigate homology and conservation among different species, comparison of the human *AF9* was conducted, revealing that *AcAF9* shares 42.3% homology with the coding region of *MLLT3* (Fig. 3D). Amino acid sequence alignment revealed four segments with high homology, including notably the YEATS functional domain (44-66 aa) of AcAF9 that is highly similar to the amino acid sequence of human AF9 (MLLT3), with the VEKVV motif being identical (Fig. 3E).

### *AF9* inhibits viral replication by positively regulating the innate immune response of bee larvae

**Fig 4:**
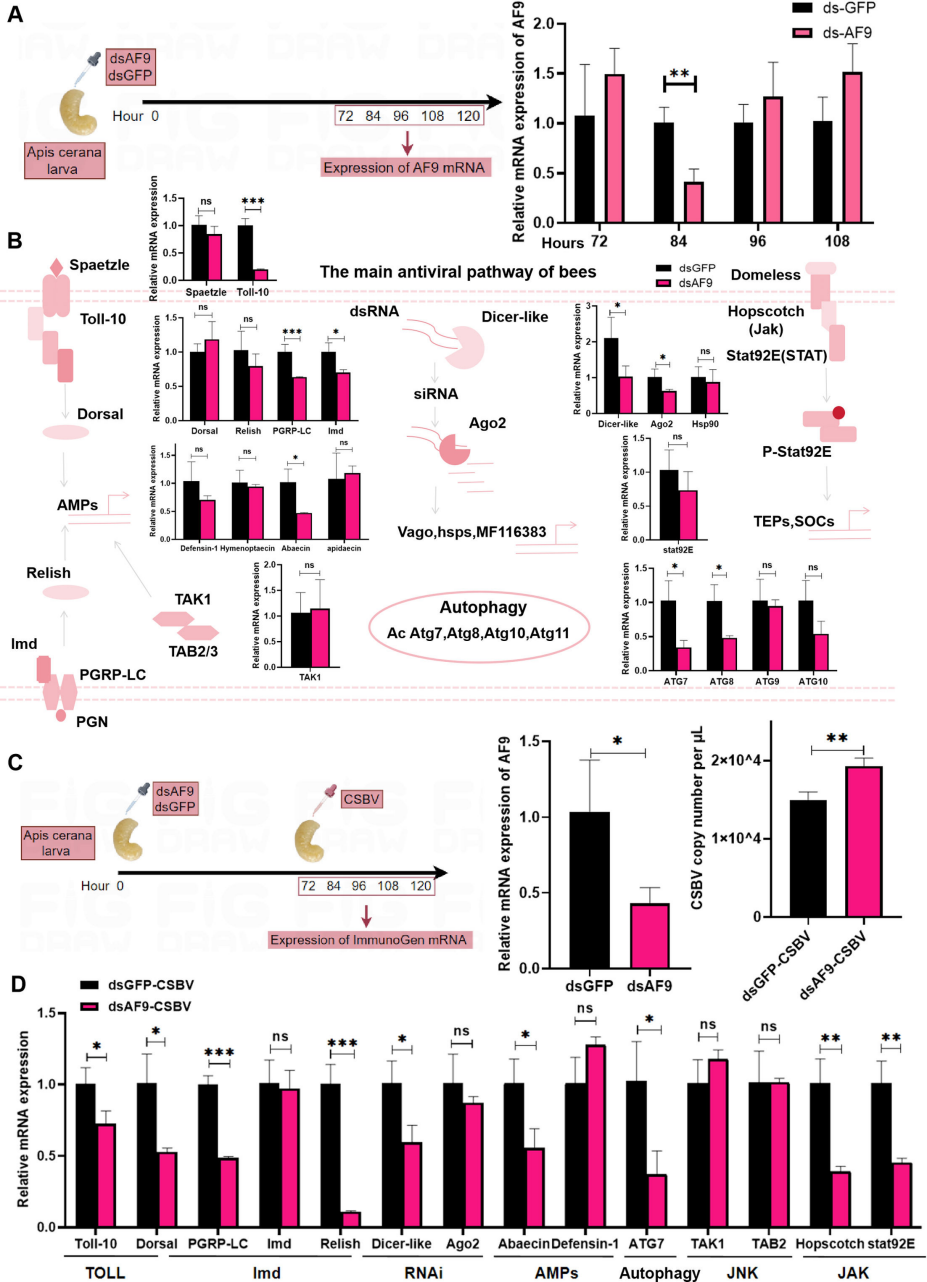
The regulatory role of *AF9* in the innate immune responses of bees and replication of CSBV. (**A**) Expression of *AF9* at 72, 84, 96, and 108 hours after injection of post-double-stranded AF9 (dsAF9) and double-stranded green fluorescent protein (dsGFP) in *Apis cerana* larvae. (**B**) The expression of major genes involved in the immune response pathways TOLL, IMD, RNAi, JNK, Autophagy, and Jak-STAT was analyzed using qPCR after the knockdown of *AF9* in *Apis cerana* larvae. Student’s *t*-tests were used to compare differences in expression in the double-stranded green fluorescent protein (dsGFP) and double-stranded *AF9* (dsAF9)-treated groups. (**C**) After feeding dsRNA (dsAF9), *AF9* expression was inhibited, and CSBV infection ensued. The dsGFP group was used as a control to detect CSBV copies and the expression of key immune genes in the innate immune pathway (**D**) .Statistical analysis of grouped comparisons was evaluated with Student’s *t*-tests (*P < 0.05, **P < 0.01, ***P < 0.001). Data shown are means ± SD, *n* = 5.

To explore whether *AF9* regulates CSBV replication, the expression of the host *AF9* was first repressed by feeding dsRNA (dsGFP/dsAF9) to the host. After testing at multiple time points, samples collected 84 hours after dsRNA feeding were selected to detect gene expression (Fig. 4A). To assess the regulatory role of *AF9* on the host’s innate immune response, transcription levels of genes within the IMD, TOLL, JNK, RNAi, autophagy, and Jak-STAT signaling pathways were investigated. The IMD, TOLL, RNAi, and autophagy pathways were positively regulated by *AF9* (Fig. 4B). Based on these results, a viral infection was initiated after dsRNA feeding (Fig. 4C) to investigate whether *AF9* regulates viral replication. When *AF9* was knocked down, the number of viral copies significantly increased compared to the control group, confirming that *AF9* exerts a significant antiviral function against CSBV replication (Fig. 4C). To determine the impact of *AF9* on the innate immune response during viral infection, the expression of key genes in various immune pathways was evaluated at the same time point. *AF9* exhibited a stronger and broader regulatory role in immune responses relative to before viral infection (Fig. 4D). The IMD and TOLL pathways remained the primary regulatory targets of *AF9*. However, the Jak-STAT antiviral pathway was not regulated by *AF9* prior to viral infection and exhibited significant downregulation after *AF9* knockdown during viral infection. Immune suppression caused by the downregulation of *AF9* is likely a key reason for significantly increased CSBV replication.

### CSBV inhibits *AF9* expression by promoting AcMETTL3-mediated m6A modifications

**Fig 5:**
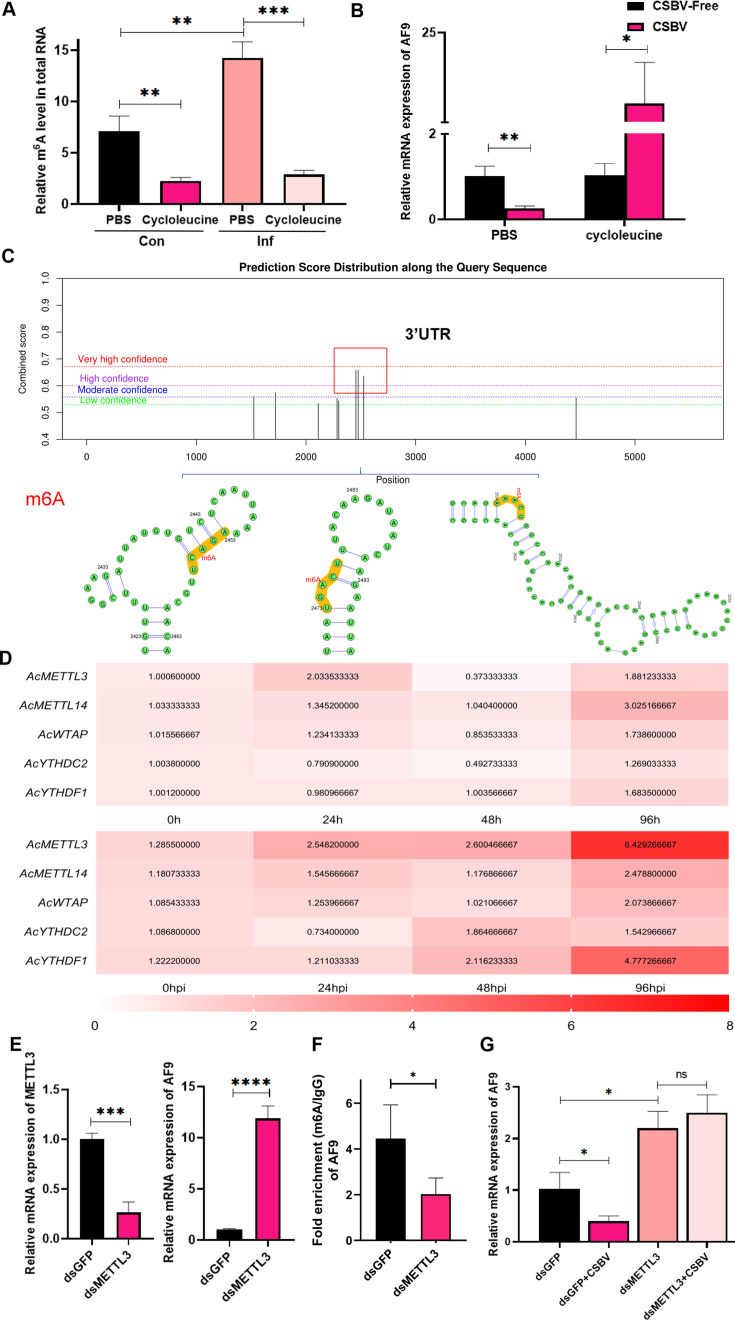
CSBV inhibits the expression of *AF9* through m6A modification of AcMETTL3. (**A**) m6A quantitative analysis was used to evaluate the percentage of m6A content in bees fed with PBS or cycloleucine to non-infected and infected (CSBV) larvae. (**B**) qPCR evaluation of *AF9* mRNA expression in non-infected and infected CSBV larvae after feeding with PBS or cycloleucine. (**C**) Bioinformatic prediction of the m6A site of *AF9*. (**D**) qPCR detection of differentially methylated genes (*AcMETTL3*, *AcMETTL14*, *AcWTAP*, *AcYTHDC2*, and *AcYTHDC3*) at different time points (0, 24, 48, and 96 hours/hpi) in non-infected and infected CSBV larvae. (**E**) RT-qPCR quantification of *AF9* expression in *AcMETTL3*-silenced (fed dsMETTL3) and GFP-silenced control *Apis cerana* larvae (fed dsGFP). (**F**) m6A levels of *AF9* mRNA in *AcMETTL3*-silenced (dsMETTL3-fed) and dsGFP-silenced (dsGFP-fed) *Apis cerana* larvae. m6A-modified mRNAs were immunoprecipitated using an m6A antibody and then quantified using qPCR with *AF9* primers. IgG was used for the negative control and enabled the calculation of the relative m6A levels. (**G**) *Apis cerana* larvae were fed dsGFP or dsMETTL3 for 36 hours and then infected with CSBV for 48 hours. Expression of *AF9* mRNA was measured with RT-qPCR. Statistical analysis of grouped comparisons was evaluated with Student’s *t*-tests (*P < 0.05; **P < 0.01; ***P < 0.001; NS, not significant). Data shown are means ± SD, *n* = 5.

The above results demonstrate that CSBV infection suppresses *AF9* expression, thereby causing immune suppression in bee larvae, which ultimately leads to enhanced viral replication. However, the mechanism by which CSBV suppresses *AF9* expression remained unclear, and in particular, whether *AF9* functioned similarly to *MLLT3* in regulating hematopoiesis, aside from regulating immune responses. MeRIP-seq data revealed that *AF9* mRNA expression decreased due to enhanced m6A modification following CSBV infection (Fig. 2B), suggesting that *AF9* may be regulated and suppressed by methylation induced by the virus. To validate this hypothesis, we first treated larvae with cycloleucine to inhibit m6A modification, while the control group was fed phosphate-buffered saline (PBS) (without using highly toxic dimethyl sulfoxide [DMSO]). The overall m6A modification levels were then evaluated in both the CSBV-infected and non-infected groups, revealing that CSBV infection (in both groups fed PBS) led to increased overall m6A modification levels in larvae, while also confirming that feeding cycloleucine inhibited larval m6A modification (Fig. 5A). The expression of *AF9* was consequently evaluated, revealing that larvae with normal m6A modification suppressed *AF9* expression after CSBV infection, while larvae with m6A modifications removed the lost repression of *AF9* expression upon virus infection (Fig. 5B). These results demonstrate that CSBV infection leads to increased m6A modification levels that then suppress *AF9* expression through m6A modification.

To explore the modification mechanism, SRAMP (an online prediction site for m6A modification sites, http://www.cuilab.cn/sramp/) was used to analyze and predict the m6A modification sites of *AF9* mRNA (21). Three high-confidence m6A modification sites were identified in the 3′ UTR region of *AF9* mRNA (Fig. 5C). Methylation modification requires the action of methyltransferases, so the expression of several important known methyltransferases from bees was evaluated during CSBV infection to identify the key enzymes responsible for m6A modification of *AF9* mRNA, including *AcMETTL3*, *AcMETTL14*, *AcWTAP*, *AcYTHDC2*, and *AcYTHDC3*. Infection of CSBV led to the gradually increased expression of two methylation genes: *AcMETTL3* and *AcYTHDC3* (Fig. 5D). Several studies have confirmed that METTL3 is a key methyltransferase in eukaryotes (22, 23). Consequently, the relationships between METTL3 and methylation modification mediated by CSBV were investigated in the context of *AF9* mRNA expression. dsRNA was fed to bee larvae to silence AcMETTL3, revealing that *AF9* expression significantly increased compared to the GFP silencing control (Fig. 5E). MeRIP methods were then used to compare enrichment levels of m6A modifications on *AF9* mRNA between bee larvae fed dsMETTL3 and those fed dsGFP as controls (Fig. 5F). The number of *AF9* mRNA fragments enriched using m6A antibodies in bee larvae fed dsMETTL3 significantly decreased compared to the dsGFP silencing control group, indicating that AcMETTL3 catalyzes m6A modification of *AF9*.

Finally, we investigated whether CSBV inhibits *AF9* by promoting the expression of AcMETTL3. Honeybee larvae were fed dsMETTL3 and subsequently infected with CSBV, followed by collecting samples 48 hours later to detect *AF9* expression. Silencing AcMETTL3 resulted in CSBV losing its ability to suppress *AF9* expression compared to the dsGFP group (Fig. 5G). Thus, CSBV infection promotes overall modification levels of m6A, and CSBV inhibits the expression of *AF9* through m6A modification, specifically by targeting modification of *AF9* mRNA via AcMETTL3 activated after CSBV infection.

### Maintenance of normal components and functional verification of hemolymph in AF9 bee larvae

**Fig 6:**
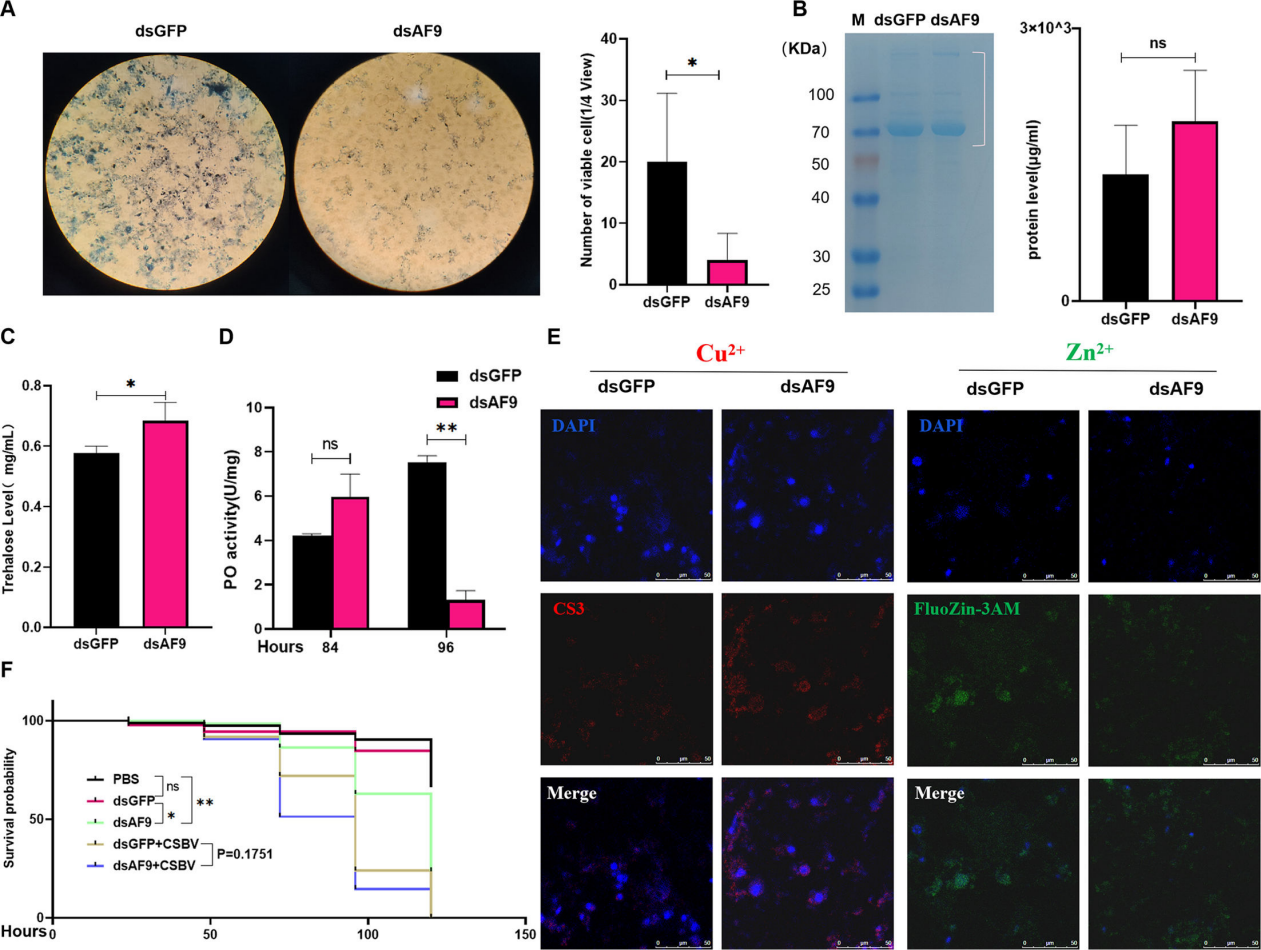
*AF9* is critical for maintaining normal structures and functions of bee larval hemolymph. (**A–E**) *Apis cerana* larvae were fed dsGFP or dsAF9 for 24 hours, the same volume of hemolymph was extracted from larvae and then observed after staining with 4% trypan blue, followed by enumeration of living cells (**A**). Detection of total protein content (**B**), total trehalose content (**C**), phenol oxidase activity (**D**), and the concentrations of Cu^2+^ and Zn^2+^ (**E**) in blood plasma or lymph. (**F**) *Apis cerana* larvae were fed dsGFP or dsAF9 for 36 hours and then infected or not infected with CSBV for 48 hours to detect larval survival rates. Statistical analysis of grouped comparisons was evaluated with Student’s *t*-tests (*P < 0.05; **P < 0.01; ***P < 0.001; NS, not significant). Data shown are means ± SD, *n* = 5.

Phylogenetic and amino acid homology analysis suggested that the bee-derived *AF9* gene may have functional similarities with human *AF9* (MLLT3). After staining the hemolymph of bee larvae fed dsRNA (dsGFP/AF9) using 4% trypan blue, viable cells were counted. To ensure the uniformity of experimental samples, the hemolymph of three larvae per group was evenly mixed, followed by the use of 50 µL for staining. Microscopic observation and statistical analyses compared to the GFP silencing control revealed that silencing *AF9* resulted in a significant reduction in viable cells within larval hemolymph (Fig. 6A). Using the same sample collection methods, the total protein and trehalose levels were measured in the hemolymph of the two groups, revealing the lack of significant difference in total protein levels between the two groups and only changes in the expression levels of some individual proteins. However, total trehalose levels significantly increased in the dsAF9-fed group (Fig. 6B and C). Subsequently, concentrations of important metal ions in the hemolymph were also evaluated using fluorescent indicators (CS3 for Cu^2+^ and FluoZin-3AM for Zn^2+^) after pre-treatment (24, 25), followed by investigation with laser confocal microscopy. The concentration of copper ions in the hemolymph of larvae silenced for *AF9* increased compared to the GFP silencing group, while the concentration of zinc ions decreased (Fig. 6E).

Reduced expression of *AF9* in bee larvae leads to decreased numbers of viable cells in their hemolymph and alters the concentrations of its primary components, including proteins, sugars, and metal ions. The effects of *AF9* on the activity of phenoloxidase in hemolymph were detected using colorimetric methods. After knocking down *AF9*, the gene-silencing effect of dsRNA gradually appeared, and the activity of phenoloxidase significantly decreased (Fig. 6D). Similarly, survival rate analysis indicated that the silencing of *AF9* resulted in a higher mortality rate in the absence of CSBV infection. After infection with CSBV, larvae with silenced *AF9* exhibited a higher mortality rate compared to the silenced GFP group (Fig. 6F).

### Antiviral potential of AF9 in *Apis cerana* and *Apis mellifera*

**Fig 7:**
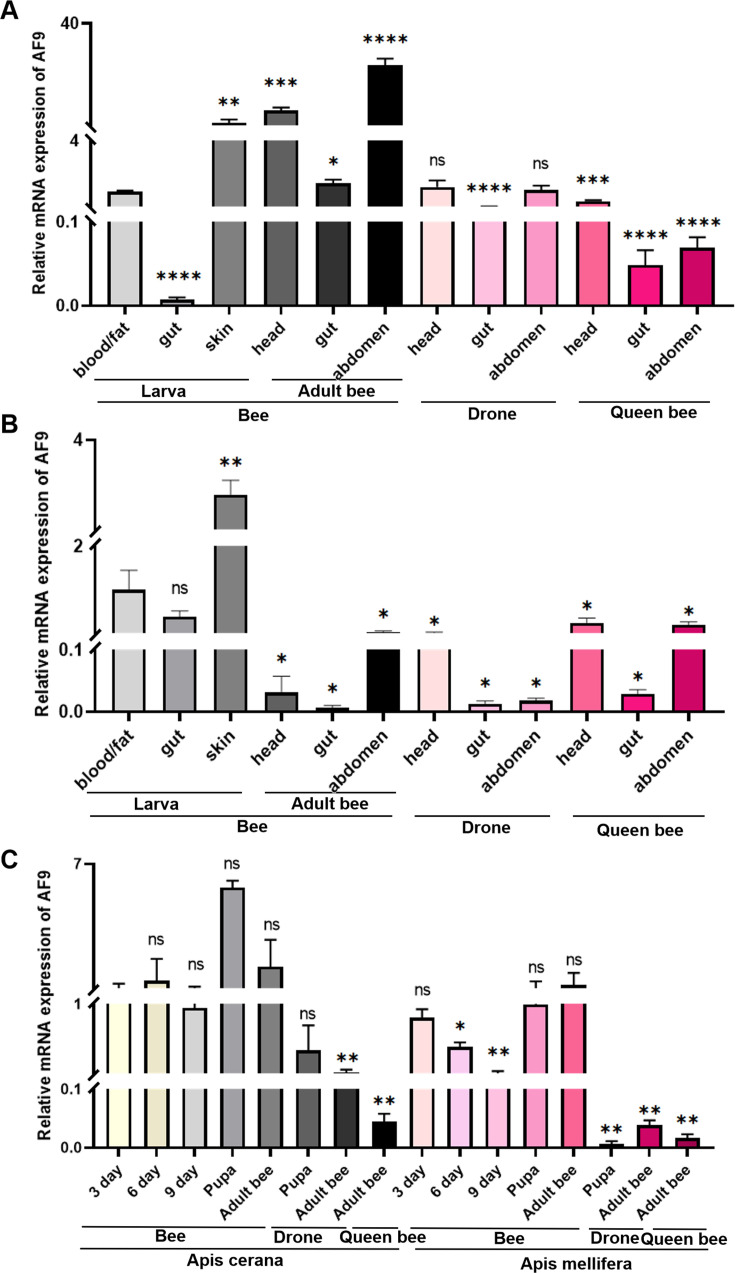
Spatiotemporal expression of *AF9* in *Apis cerana* and *Apis mellifera*. (**A**) Spatial expression of *AF9* in hemocytes and fat bodies, guts, skins (larvae and worker bees), pupae, heads, midguts, and abdomens (adult bees, worker bees, drones, and queen bees) of *Apis cerana* and *Apis mellifera* (**A and B**). (**C**) Total RNA was extracted from the 3rd, 6th, and 9th instar larvae, 1 day pupa, 1 day adult bee (worker bees, drones, and queen bees) of *Apis cerana* and *Apis mellifera*, respectively. Quantitative real-time PCR (qPCR) data were normalized to expression of actin in each treatment. Statistical analysis of grouped comparisons was evaluated with Student’s *t*-tests (*P < 0.05; **P < 0.01; ***P < 0.001; NS, not significant). Data shown are means ± SD, *n* = 3.

To further explore the antiviral capacity of *AF9*, the spatiotemporal expression of *AF9* in the Asian honeybee and *Apis mellifera* was investigated. In mature Asian honeybees, the expression of *AF9* in worker bees was higher than in drones and queens. During the larval stage of worker bees, *AF9* expression in the gut was lower than in the hemolymph, fat body, and cuticle (Fig. 7A). In contrast to the Asian honeybee, the expression of *AF9* in mature *Apis mellifera* was lower than in the larval stage, with the highest expression of *AF9* observed in the cuticle during the larval stage (Fig. 7B). Thus, *AF9* expression at different developmental stages in the Asian honeybee was generally higher than in *Apis mellifera*. Specifically, the expression of *AF9* in Asian honeybee larvae was relatively high, peaking during the pupal stage before gradually decreasing. In *Apis mellifera* larvae, *AF9* expression progressively decreased with age, thereby significantly increasing after pupation and reaching lower levels in adult drones and queens (Fig. 7C).

**Fig 8:**
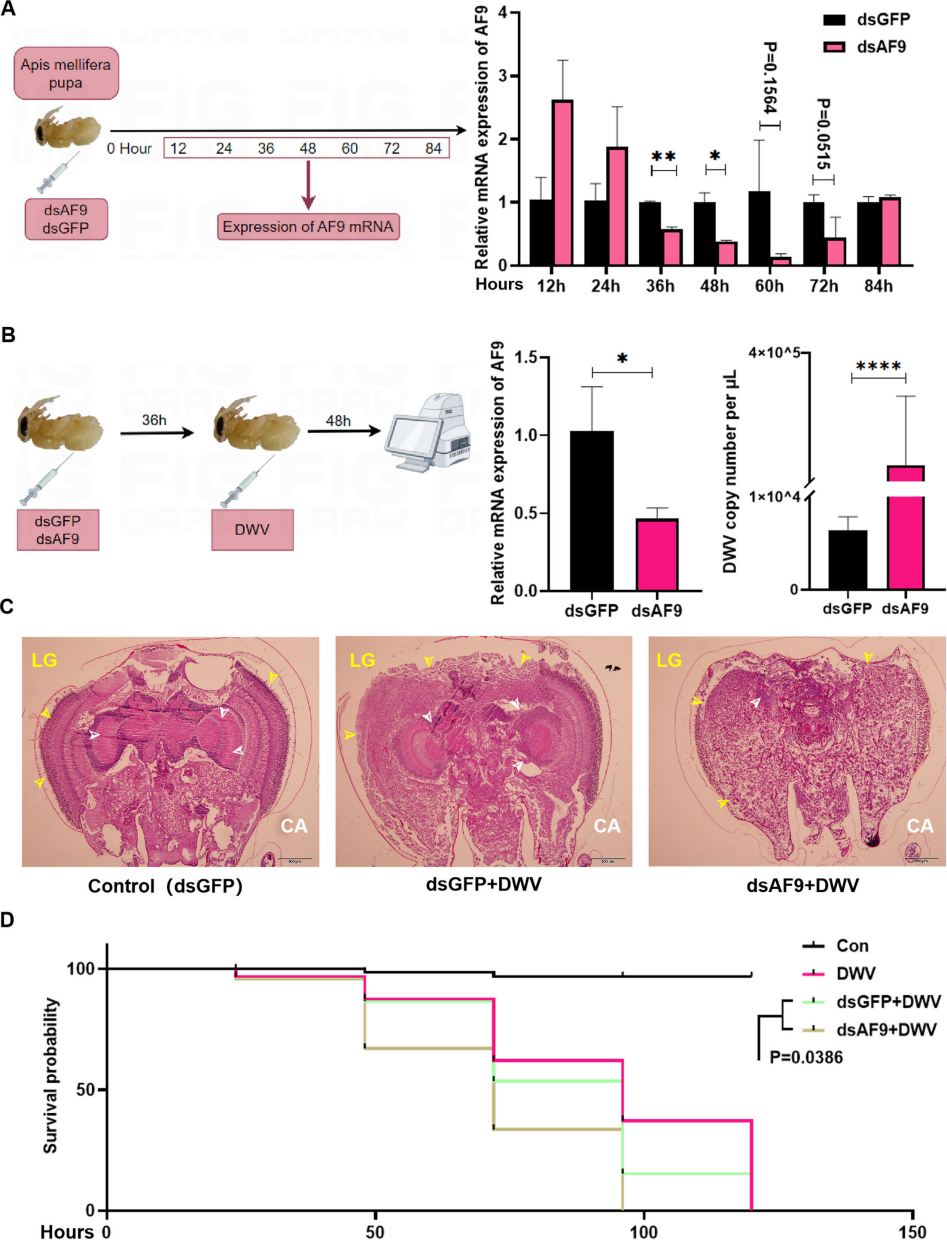
*AF9* inhibits DWV infection in *Apis mellifera* pupae. (**A**) Expression of *AmAF9* 12, 24, 36, 48, 60, 72, and 84 hours after injection of post-double-stranded *AmAF9* (dsAF9) and double-stranded green fluorescent protein (dsGFP) in *Apis mellifera* larvae. (**B**) qPCR detection showing inhibited *AmAF9* expression after injection of dsRNA (dsAF9), and DWV infection (injection) was assessed with the dsGFP injection group as a control and by measuring DWV copies. (**C–D**) Brain pathological sections (the edge profiles [LG] and typical structures [mushroom body CA]) of normal bee pupae (dsGFP) and bee pupae infected with DWV after injection of dsGFP and dsAF9 for 36 hours (**C**), along with survival rate assessments (**D**). Statistical analysis of grouped comparisons was evaluated using Student’s *t*-tests (*P < 0.05, **P < 0.01, ***P < 0.001). Data shown are means ± SDs, *n* = 5.

An infection protocol for deformed wing virus (DWV) was constructed by silencing *AmAF9* through injecting dsRNA into the pupae of *Apis mellifera* and based on gene knockdown effects (Fig. 8A). After injecting dsRNA, bees were infected with the virus 36 hours later, and samples were collected at 48 hpi to measure DWV copies. Compared to the GFP-silenced pupae group, DWV copies in the *AmAF9*-silenced group were significantly higher (Fig. 8B). Furthermore, DWV infection caused roughening of the edge profiles (lamina ganglion, LG) and inward contraction of brain tissues in pupae compared to the control group, while typical structures such as the mushroom body calyx (CA) exhibited necrosis and disappeared. Compared to the virus-infected group after GFP silencing, the degree of atrophy of virus-infected pupae after *AmAF9* silencing was more pronounced, with the mushroom body CA and other brain structures completely absent (Fig. 8C). Finally, the survival rates of pupae that were silenced and infected with DWV were evaluated, showing that the *AmAF9*-silenced pupae exhibited higher mortality rates compared to the ordinary infection and silenced control groups (Fig. 8D). The above results confirm that *AF9* also plays a significant role in inhibiting DWV infection in *Apis melliferas* pupae.

**Fig 9:**
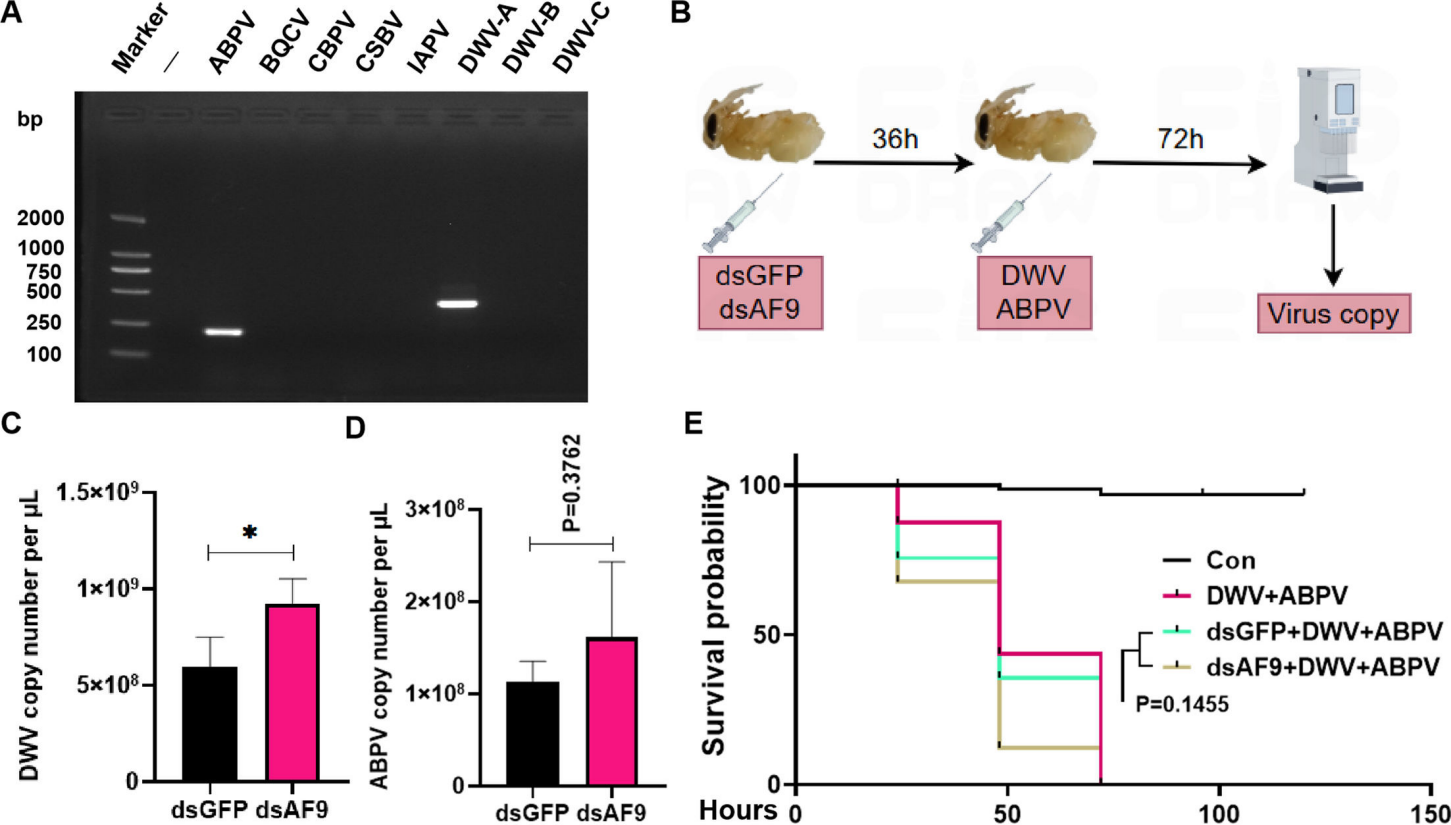
Exploring the broad-spectrum antiviral potential of *AF9*. (**A**) Detection of eight common RNA viruses of bees in a seemingly unhealthy colony. (**B–E**) qPCR detection of dsRNA injection (dsAF9) followed by infection with purified mixed viruses by injection, using the dsGFP injection group as the control. qPCR was used to detect copies of DWV and ABPV in infected bee pupae (**B–D**), as well as *Apis mellifera* pupae survival rates (**E**). Statistical analysis of grouped comparisons was evaluated using Student’s *t*-tests (*P < 0.05, **P < 0.01, ***P < 0.001). Data shown are means ± SDs, *n* = 5.

Using clinical samples of honeybees infected with mixed DWV and acute bee paralysis virus (ABPV), a mixed liquor of DWV and ABPV was isolated and purified (Fig. 9A). Subsequently, the previously designed dsRNA injection and virus infection experiments of *Apis mellifera* pupae were conducted. To obtain a better virus copy detection sensitivity, samples were collected at 72 hpi with the mixed viruses and subjected to virus copy measurement (Fig. 9B). Compared to the dsGFP injection group, DWV and ABPV copies in the *Apis mellifera* pupae were higher after silencing *AmAF9* (Fig. 9C and D). Survival rate analysis indicated that *Apis mellifera* pupae mortality in the mixed virus infection group was higher after silencing *AmAF9* (Fig. 9E). The combined results of these experiments confirmed that *AF9* plays an important role in mitigating CSBV infection in *Apis cerana* larvae and functions similarly in mitigating DWV and ABPV infection in *Apis mellifera* pupae.

**Fig 10:**
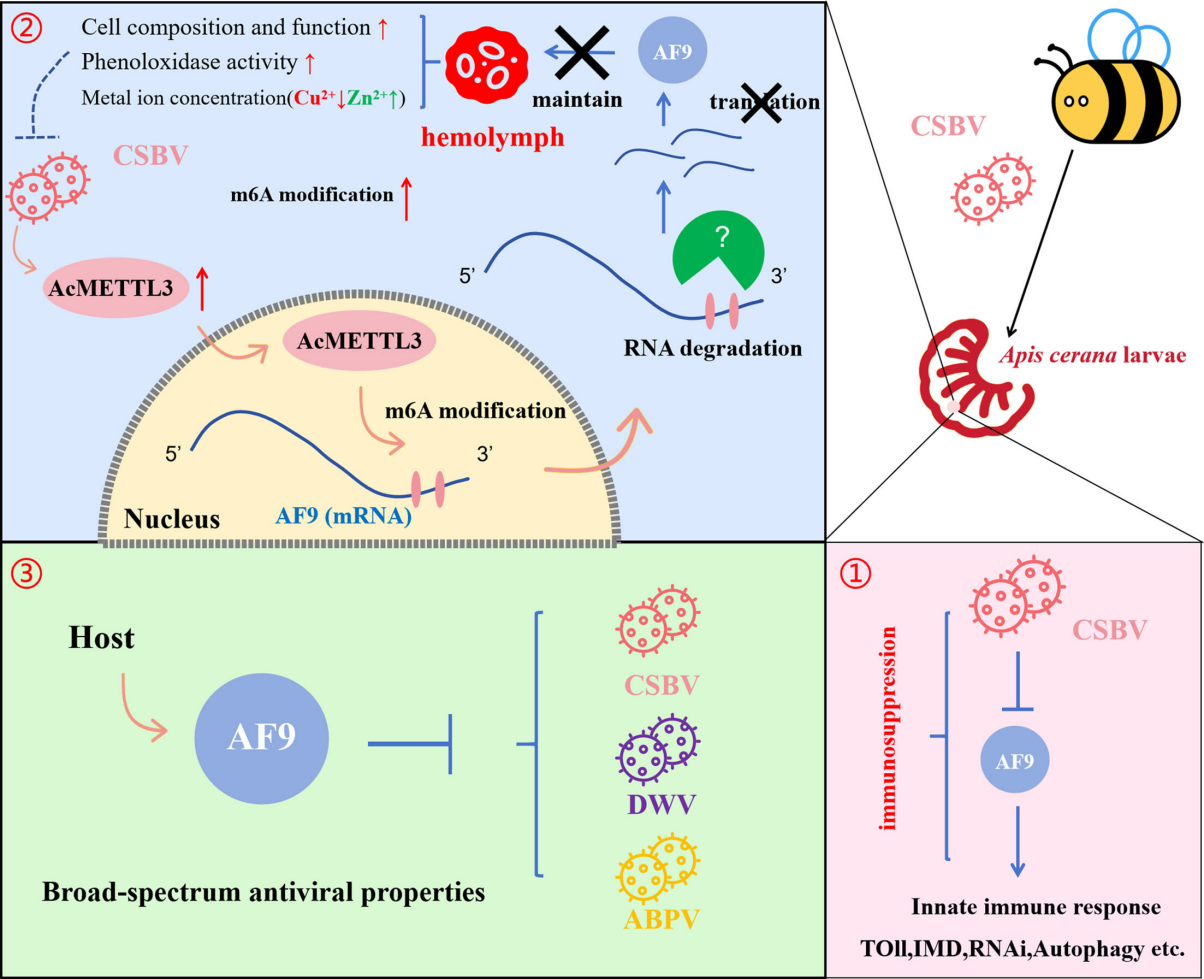
Proposed model of CSBV evasion of honeybee antiviral innate immune defenses by inhibition of *AF9*. CSBV inhibits the expression of *AF9* by mediating m6A-targeted modification, thereby blocking the innate immune responses of *Apis cerana* larvae, promoting viral replication, and demonstrating broad-spectrum antiviral capabilities within *Apis mellifera* pupae.

In summary, CSBV infection mediates an important immune suppression response in *Apis cerana* larvae by enhancing overall m6A modification in the host during a critical period of larval polar differentiation and promoting the expression of AcMETTL3 that, in turn, targets *AF9* mRNA for modification, thereby inhibiting *AF9* expression. *AF9* is a critical gene involved in maintaining the normal structures and functions of larval hemolymph, the activation of phenoloxidase, and the concentrations of key metal ions in hemolymph, in addition to the overall innate immune response. After CSBV infection, the suppression of *AF9* expression through m6A modification exacerbates further virus infection (Fig. 10). Concomitantly, *AF9* is also stably expressed in the *Apis mellifera*, serving as a broad-spectrum antiviral gene.

## DISCUSSION

The collapse of bee colonies can be caused by infections from pathogens such as viruses, bacteria, and parasites (26). The SBV is globally distributed and was the first bee virus to be discovered (27). SBV infection is deadly to bee larvae, causing the accumulation of fluid in the epidermis and leading to the formation of a cyst that prevents normal pupation. Additionally, the epidermis may turn brown and after death, the larvae may dry out into a boat-like shape. Regional differentiation and the evolution of SBV have led to the emergence of many variants, among which CSBV is one of its evolutionary derivatives. *Apis cerana* is more susceptible to CSBV than *Apis mellifera*, making it the most serious infectious disease threatening *Apis cerana*’s health (28, 29). There are relatively few studies of the pathogenic mechanisms of CSBV, primarily due to the lack of suitable cell lines for viral culture. Most research has instead been conducted on larval animal models, making the establishment of a systematic and comprehensive infection model particularly prescient. However, the difficulty of artificially rearing *Apis cerana* larvae limits standardization within an infection model. Previous studies have suggested that CSBV mainly infects larvae aged 1 to 3 days. Here, the optimal age for larval infection was comprehensively evaluated. Larvae aged 1 to 3 days were not chosen, because individuals at this age are small and fragile, making them difficult to handle, and if they were to be selected, their survival rates would be very low. Therefore, larvae aged 4, 6, and 8 days were used for experimentation. Second, our research team previously attempted to inject virus solutions into larvae using a micro-injector. Although this method ensured consistent viral dosage, the micro-injection caused trauma to larvae. Notably, physiological responses like stress can severely affect the accuracy of subsequent analyses. CSBV is classified as an intestinal virus of bees, and studies have shown that its transmission occurs through food feeding and vertical transmission (30, 31). Considering this characteristic, a natural infection of CSBV via oral feeding was mimicked in this study to initiate viral infection. To ensure a consistent viral dosage in each larva, a novel infection method was designed by mixing the virus solution with larval food (sugar water) in a 6 mL measuring cup, performing the first weight measurement and then placing the larvae into the cup for viral infection. After 6.5 hours, the larvae were removed while recovering as much liquid from the larvae back into the measuring cup as possible, followed by recording of the second weight. The first weight was subtracted from the second to determine the amount of virus ingested by the larvae. Finally, during subsequent experimental testing, samples with abnormal viral dosages were excluded, thus aligning with the true infection method of CSBV while ensuring that larvae consumed the same amount of virus. Based on these experiments, selection of 4-day-old larvae for virus infection was confirmed, and samples were collected for testing at 48 hpi, because viral copy number significantly increased at 48 hpi and pathological histology analysis verified this result. Thus, optimization of the CSBV infection model for *Apis cerana* larvae enabled an accurate and rigorous method to evaluate the viral infection of larvae.

Female honeybee larvae with the same genotype can develop into phenotypically different organisms (i.e., sterile worker bees and fertile queens) depending on conditions such as diets (32). Previous studies have shown that DNA methylation and histone modifications can establish different gene expression patterns, leading to developmental differentiation of honeybee larvae (33). Specifically, the H3K4me1 marks in 2- and 4-day-old worker bee larvae are more abundant than in queen larvae, and many genes associated with polar differentiation exhibit differential methylation modifications (32, 34). In light of these observations and the high susceptibility of 1- to 4-day-old larvae to CSBV, we sought to evaluate several questions: whether RNA modifications during the polar differentiation period of honeybee larvae also peak, whether CSBV infection impacts the most common m6A modification in RNA methylation, and what effects m6A modifications in larvae have on viral infection. To address these questions, a combined MeRIP-seq and RNA-seq analytical approach was used to explore interactions between CSBV infection and m6A modifications in larvae. Many host genes exhibited negative correlations between m6A modification levels and mRNA expression, allowing the filtering of potential functional genes for further validation. Additionally, CSBV infection led to increased overall m6A modification levels in larvae, with two known honeybee m6A modification-related genes (*AcMETTL3* and *AcYTDHC3*) exhibiting elevated expression. These results demonstrate that CSBV infection induces changes in host m6A modifications, which should be further explored in subsequent in-depth investigations.

CSBV infection has been shown to suppress *AF9* expression, and this suppressive effect is achieved through m6A modification. m6A methylation is catalyzed by a multi-protein complex known as the “writer” that comprises the subunits METTL3, METTL14, and WTAP, with METTL3 being the main catalytic subunit responsible for executing methyl group transfer. Conversely, two demethylases, FTO and ALKBH5, act as “erasers,” responsible for removing m6A modifications from mRNA. Additionally, proteins containing the YT521-B homology (YTH) domain (YTHDF1-3 and YTHDC1-2) serve as “readers” that specifically recognize m6A modifications of mRNA. The readers bind to the modified mRNA and regulate gene expression by promoting mRNA decay or modulating mRNA translation. MeRIP-qPCR enabled the identification of AcMETTL3 as the key methyltransferase gene mediating this modification process. However, this research has certain limitations. *AcMETTL3* is the catalytic gene for the methylation modification of *AF9*, but the definitive identification of the gene responsible for its recognition has not been achieved. It remains uncertain whether this key gene is another methylation regulatory gene, AcYTDHF1 (which is upregulated due to viral infection), or other methylation reader enzymes. Further systematic and comprehensive screening in subsequent studies is needed to clarify the downstream recognition proteins that regulate *AF9* methylation. In addition, *in silico* predictions suggested that the m6A modification sites are in the 3′ UTR region of *AF9* mRNA, but the specific binding region of *AF9* mRNA with AcMETTL3 was not identified and should be evaluated in future studies. Low-age larvae are in a stage of extreme differentiation. Whether the methylation modifications that occur in the larvae during this stage are conducive to CSBV infection and whether this is a reason for the high susceptibility of larvae to CSBV at this stage will be a focus of our future research.

Bees do not have a closed circulatory system like mammals, wherein their body cavities are filled with a mixture of open hemolymph and fat. Their immune systems are also much simpler than those of mammals. In addition, the organizational structure of bee larvae is simpler than in adult bees, with a layer of outer skin enveloping the cavity filled with hemolymph and fat, while the center comprises undeveloped intestinal tissues and other glandular tubes. Cell-mediated immunity mediated by hemolymph can engulf and encapsulate pathogens, while the phenoloxidase system within it produces melanization and reactive oxygen species, thereby stimulating the entire humoral immune response. Consequently, before a complete intestinal structure forms in larvae, hemolymph is the core mechanism by which the host defends against external pathogen infections. Initially, we observed that *AF9* knockdown leads to inhibition of the innate immune responses in bee larvae, alongside significant increases in viral copies, thus demonstrating that *AF9* is a positive immune regulatory factor for the host. The regulatory effects of *AF9* influence the primary immune pathways for antiviral defense in bees, including the RNAi, TOLL, IMD, JNK, Jak-STAT, and autophagy pathways.

The mechanism underlying the effects of *AF9* on the innate immune response of *Apis cerana* larvae was the focus of subsequent research. Previous studies have shown that MLLT3 plays a critical role in regulating artificial hematopoiesis (35). The YEATS domain is a conserved segment of MLLT3 that exerts significant transcriptional regulatory functions and is one of the key functional domains by which MLLT3 exerts physiological roles (36). Homology and functional analysis of human AF9 (MLLT3) and AcAF9 revealed that the YEATS domain sequences of AcAF9 and MLLT3 were highly similar. Extraction of hemolymph after knocking down *AF9* revealed that reduction of *AF9* led to decreased numbers of live cells in larval hemolymph that would significantly impact host cellular immunity. Concomitantly, the activity of phenoloxidase decreases, which would affect melanization and the production of reactive oxygen species, even inhibiting the entire humoral immune response. Comparative deduction allowed the initial identification of how *AF9* positively regulates immunity. Combined analysis of the immunity and physiology of insects and mammals revealed that AF9 functions similarly to mammalian MLLT3 by influencing hematopoiesis. However, it should be noted that the total amount of protein and trehalose was nearly unaffected by *AF9*, while the concentrations of copper and zinc ions in hemolymph exhibited differences. Increasing evidence suggests that intracellular metal ions can influence pathogen infections, with zinc and copper ions being important research targets (37–40). Moreover, zinc and copper ions may be intimately associated, as they sometimes share the same transport proteins, indicating potential competition between them, similar to results described in this study (41). Therefore, investigating the impact of trace metal ion concentrations in hemolymph on host immunity and antiviral responses is an important research direction for the future. Reduced *AF9* expression leads to increased copper ion concentration and decreased zinc ion concentrations. Nevertheless, the mechanisms underlying these differences and how changes in the concentrations of trace metal elements in hemolymph affect immune responses and viral infections remain unclear and warrant additional studies.

Spatiotemporal analysis of *AF9* expression in *Apis cerana* and *Apis mellifera* revealed that *AF9* is expressed in different bee species, ages, and tissues. It was also found in the genetic evolution analysis that the phylogenetic branch of AF9 in *Apis mellifera* was more similar to that of MLLT3, suggesting that it may be involved in broad-spectrum antiviral activities. Some common viruses of *Apis mellifera* primarily spread through Varroa mite infestations of bee pupae. Consequently, an infection model using *Apis mellifera* pupae was established to assess the *AF9* broad-spectrum antiviral properties (42). Reduced *AF9* expression also led to significantly increased levels of DWV copies and mortality rates, in addition to exacerbated field mixed infections. The influence of *AF9* on DWV may also be mediated by m6A modification, and this is a subject that requires further verification. In addition, the drone, queen, and workers are different castes, and m6A modification would cause different changes. Therefore, the regulatory role of m6A modification in the replication of bee viruses by the queen will be an important direction of our future research. This may provide new insights into the vertical transmission mechanism of bee viruses within bee colonies. Thus, it is particularly important to continue exploring potential application value of modulating *AF9* expression. Bees are social insects, and the entire bee colony is essentially derived by the queen’s reproduction. This characteristic can be leveraged and combined with mRNA vaccine technology to introduce an *AF9* transcriptional enhancer gene into the eggs of the queen bee, thereby successfully rearing a queen that establishes a colony with high *AF9* gene expression and possible higher resistance to bee viruses. Nevertheless, biosafety issues require consideration, and the broader application potential of this gene will be explored in our future research.

This study is among the first to elucidate how m6A modifications mediated by bee viruses inhibit immune responses by targeting and suppressing important host immune-positive regulatory factors, thereby facilitating infection of the host. The potential application of m6a modifications was also preliminarily explored as a broad-spectrum antiviral target against bee viruses. This study provides new insights into the pathogenic mechanisms of bee viruses, describes novel methods for preventing and controlling CSBV, and further elucidates our overall understanding of CSBV.

## MATERIALS AND METHODS

### *Apis cerana* larvae and rearing

The *Apis cerana* colonies used in the study were sourced from a local apiary in Jinzhou, Liaoning Province, China. To obtain larvae of the desired age for experimentation, a healthy queen was removed from the colony and confined to lay eggs on the brood frame for 12 hours. After 48 hours, a brood frame containing 2-day-old larvae was taken from the colony. The 2-day-old larvae were then individually transferred to a 48-well plate. The 48-well plate was placed in an incubator set at 32°C ± 1°C and a relative humidity of 75% ± 5%, with artificial larval feed provided to the larvae. Feed was replaced daily until the larvae reached the required age for the experiments. The larval feed used in the study contained the following elements: 2 g royal jelly, 3 g *Apis cerana* honey, 8.7 mL sterile water, 0.6 g glucose, 0.6 g fructose, and 0.1 g yeast.

### Viruses

To purify the CSBV, 3- to 4-day-old *Apis cerana* larvae that showed visible disease symptoms (formation of fluid sacs) were collected. The individuals were mixed with 5 mL of sterile PBS in a sterile mortar and ground to a homogenate. The homogenized mixture was centrifuged at 8,000 rpm for 30 minutes at 4°C. The supernatant was then filtered twice through a 0.22 μm filter membrane to remove tissue debris. The virus was extracted from the honeybee larvae samples infected only with CSBV by filtering the liquid through RT-PCR detection to exclude other viral infections. The collected viral solution was then further purified by cesium chloride (CsCl) gradient centrifugation and dialyzed against PBS to remove CsCl (1,100 rpm, 10 minutes, 4°C; 5,000 rpm, 20 minutes, 4°C; 8,000 rpm, 30 minutes, 4°C; 35,000 rpm, 1 hour, 4°C). Finally, the presence of other major bee viruses in the purified virus was identified using PCR, further confirming it as a single virus (Fig. S1 and the primer sequence for RT-PCR and PCR detection in Tables S1 and S2). The purified viral solution was stored at −80°C for later use.

DWV and a mixture of DWV/ABPV were extracted from the pupae of western honeybees, and the purification method was the same procedure as mentioned above.

### Viral infection

All bee larvae and pupae used in this study were tested for the presence of the DWV, the chronic bee paralysis virus (CBPV), the Israeli acute paralysis virus (IAPV), and the ABPV to evaluate viral infection status.

*Apis cerana* larvae were infected with CSBV by being immediately transferred from the honeycomb to a 48-well plate and placed in a bee incubator, where they were fed twice daily. To initiate infection, a 6 mL cup containing 1 mL of food mixed with 100 μL/mL of virus solution was weighed and recorded. The larvae were then placed into the solution cup, and after feeding for 7 hours, the larvae were removed and any virus-laden liquid adhering to their surfaces was returned to the cup. The cup was weighed again, and the difference in weight between the two measurements indicated the amount of virus ingested by the bees. Prior to testing, any larvae with significant weight discrepancies were removed to ensure a consistent infection dosage for the remaining experiments.

To infect *Apis mellifera* pupae with DWV, the pupae were held in place with ophthalmic forceps, and a micro-syringe was inclined at a 45° angle to inject 0.8 μL of the purified virus solution into the side of the second abdominal segment of bee abdomens. After injection, a small amount of petroleum jelly was applied to the wound surface, and the *Apis mellifera* pupae were returned to the constant temperature incubator for further cultivation and use in subsequent experiments.

### RNA extraction and m6A MeRIP

The total RNA was extracted using TRIzol (Invitrogen), and the concentration and integrity of the extracted RNA were measured using a Qubit RNA HS Assay and an Agilent 2100 Bioanalyzer (Agilent Technology), respectively. For the MeRIP experiment, approximately 20 μg of the total RNA from each sample was fragmented using a 10× RNA Fragmentation Buffer (Invitrogen) by incubating in a preheated thermal cycler for 10 minutes at 70°C. The fragmented RNA was pelleted using ethanol precipitation. Protein A and protein G magnetic beads were washed twice with an IP buffer (150 mM NaCl, 10 mM Tris-HCl, pH 7.5, 0.1% IGEPAL CA-630 in nuclease-free H_2_O) prior to incubation with 5 μg of m6A antibody (Synaptic Systems) at 4°C for 2 hours. After two washes with the IP buffer, the antibody–bead complexes were resuspended in 500 μL of the IP reaction mixture that included the fragmented total RNA and incubated for 4 hours at 4°C. The immunoprecipitated m6A RNA with protein A/G magnetic beads was then washed three times using the IP buffer for 10 minutes each at 4°C. Finally, the bead complexes were resuspended in 100 μL of m6A competitive elution buffer with continuous shaking for 1 hour at 4°C. The supernatant containing the eluted m6A RNA was collected and purified using phenol:chloroform:isoamyl alcohol (125:24:1).

### m6A-seq assay and data analyses

MeRIP libraries using the eluted RNA were constructed using the SMARTer Stranded Total RNA-Seq Kit version 2 (Takara/Clontech) according to the manufacturer’s protocol. All libraries were analyzed using an Agilent 2100 Bioanalyzer (Agilent Technologies) and quantified using real-time PCR prior to sequencing. Low-quality bases were trimmed using trimmomatic (version: 0.38). The options and parameters used to filter raw reads were ILLUMINACLIP:MeRIP-PE.fa: 2:30:10:1:true, SLIDINGWINDOW: 30:15, AVGQUAL: 15, LEADING: 15, TRAILING: 15, and MINLEN: 30. Clean reads were mapped to the genome assembly of *Apis mellifera* (Amel_4.5) using HISAT (version 2.1.0) with default parameters. The alignment file (SAM) was transformed to a BAM file and filtered using the following steps: (i) only unique, properly aligned reads were kept; (ii) reads with low MAPQ (<30) were removed; and (iii) reads mapped to blacklist regions were removed. The R package exomePeak (version: 2.1.2) was used to call peaks and detect differential peaks from filtered alignment files with the following parameters: WINDOW_WIDTH = 200, SLIDING_STEP = 30, FRAGMENT_LENGTH = 150, DIFF_PEAK_ABS_FOLD_CHANGE = 2, and FOLD_ENRICHMENT = 2. The annotation file used to annotate the peaks was downloaded from the University of California Santa Cruz (UCSC). Only peaks with a false discovery rate (FDR) of <0.05 and a fold change of ≥2 were identified as being significantly differential. The gene expression calculation was performed using StringTie software with default parameters. The gene expression profiling was based on the number of reads. Transcripts per million mapped read values were used to estimate the expressed values and transcript levels. DESeq2 was selected to identify the differentially expressed genes (DEGs). Genes with an adjusted *P* value (padj) of <0.05 and an absolute log2 (fold change) of >1 were considered to be DEGs. For the correlation analysis of the metagene profiling/plots, we applied the metagene2 software (http://mirrors.nju.edu.cn/bioconductor/packages/3.10/bioc/vignettes/metagene2/inst/doc/metagene2.html).

### Inhibitor analysis

Cycloleucine (CAS: 52-52-8) is also known as 1-amino-cyclopentanecarboxylic acid and is used as a methylation-modifying inhibitor. A total of 0.05 µg of cycloleucine was dissolved in 10 μL of ultrapure water, and 1 μL of solution (including 0.005 μg of cycloleucine) was fed to each bee larva by adding it to the sugar water feed. The control group was fed only sugar water.

### Synthesis and delivery of dsRNA

Double-stranded RNA of *AF9* and *METTL3* genes of *Apis cerana* were designed and synthesized (sequences are shown in Table 1). To feed dsRNA to honeybee larvae, the synthesized dsRNA stock solution was dissolved in RNase-free water at a ratio of 1:100 to prepare the working solution. The working solution was mixed with larval feed, and each larva was fed 100 μL. After feeding for 12 hours, the larvae were transferred to a new 48-well plate for the next experiment.

**TABLE 1:** Primers for the dsRNA synthesis

dsRNA sequence	dsForward	dsReverse
dsAF9	TAATACGACTCACTATAGGGGCAGAAATGCCAGAAAGGGA	TAATACGACTCACTATAGGGTCTTCCGAAAACACTGGTGGT
dsMETTL3	TAATACGACTCACTATAGGGCCTGCTCTTCAAGATGAAGG	TAATACGACTCACTATAGGGCATTGAGTTTCCATCCGGAT
dsGFP	TAATACGACTCACTATAGGGGCGAGGGCGATGCCACCTAC	TAATACGACTCACTATAGGGCACGCTGCCGTCCTCGATGT

dsRNA was injected into pupae by dissolving the synthesized and dissolved dsRNA stock solution in RNase-free water at a ratio of 1:100 to prepare the working solution. An ophthalmic tweezer was used to hold the pupa in place, followed by injection of 1 μL of dsRNA into the side of the second abdominal segment of each pupa using a micropipette. After injection, a small amount of petroleum jelly was applied to the wound surface.

### Quantitative real-time PCR (qRT-PCR)

qRT-PCR was conducted by collecting three or five individual larva per group, followed by RNA isolation using TRIzol (TransGen Biotech, ET111-01-V2) according to the manufacturer’s protocol. The concentration and purity of RNA samples were evaluated using a Nanodrop 2000 spectrophotometer (Thermo Fisher Scientific, USA). Reverse transcription was performed using the First Strand cDNA Synthesis Kit (Thermo Fisher Scientific, USA). The QuantStudio3 instrument (Thermo Fisher Scientific, USA) was then used to conduct thermal cycling, including a 50°C denaturation stage for 2 minutes, a 95°C denaturation stage for 2 minutes, a 95°C denaturation stage for 15 seconds, and a 60°C annealing/extension stage for 1 minute, for a total of 40 cycles. A melt curve was generated after each run, which included 95°C for 15 seconds and 60°C for 1 minute, over an increment of 0.3°C until reaching 95°C for 15 seconds. The relative expression of transcripts was evaluated using the 2^-ΔΔCT^ method. Each sample was technically replicated three times on the same plate. Virus copy detection pre-treatment was conducted as described above, with thermal cycling conditions including 94°C denaturation for 30 seconds, followed by amplification at 94°C for 5 seconds, and 59°C for 30 seconds for 40 cycles. RNA-free water was used as the template for negative control reactions, with each sample technically replicated three times on the same plate. The primers and probes used in the PCRs are indicated in Tables 2 and 3.

**TABLE 2:** Primer sequences for qPCR

Gene ID	GenBank accession	Forward primer	Reverse primer
β-actin	NM_001185145.1	ATGCCAACACTGTCCTTTCTGG	GACCCACCAATCCATACGGA
METTL3	XM_017067144.3	AGTGGAGAAACTGAAGTGTTGTA	GCCTGATGAAGAATCCGTTTGT
METTL14	XM_017048454.2	TGTGGTGACCAGTGATGATGA	ACCACTTTTACCACCTGCCC
WATP	XM_062081828.1	GCGTGTCAGTATATCAGGCAAAT	ATCATTGCACTCCTCTGCCA
YTHDC2	XM_017057485.3	AGCTTTGTTTTGTGGACCAGC	CACTTTCTGGTACAGATTCAGC
YTHDF	XM_017049177.3	CGAGGAGGATTTGGCACTGT	ACGAATTCTCTTCACAGCCAT
LOC108000203	XM_017060427.3	AGAAGTTGCACGAGTCCGAG	TCGAAACAGTTATCTTTTCCGTTT
LOC107995138	XM_017052465.3	ACCAAACCGAAGGTTGTTGG	AGCAGCCATTTCTGCTTCCA
LOC108002262	XM_062085886.1	CCTCTCCTCCGTACGAGCTA	AAACGTTACAGCTCTCCCCG
LOC108002611	XM_062079243.1	CACCACCACGAACTGAACAAA	AAAGGCAGATTGGCTAGGGC
LOC108003260	XM_017065414.3	CGACAACTGACACGACTAGGAAA	GCGATACCGGCCTCAAAGTC
LOC107992733	XM_017048780.3	TCGGACGTGTGTTCGTAACC	ACGCATAGCATCTGCCCATC
LOC107992887	XM_017049002.3	GCTGGCGAACAAGATCAGAG	TCGCCGACGTTCTGATAACT
AF9	XM_017049815.3	AAAAGGCGGTGGTGTTCTCA	AGGAGTTCCAAATAGCTCAGCA
TOLL	XM_016911914.1	CAAATCGCGCGTTCTGTTGA	TCTGCGTTCACTGAGTGCTT
Dorsal	XM_028665862.2	AGAGATGGAACGCAGGAAAC	TGACAGGATATAGGACGAGGTAA
PGRP-LC	XM_017066696.3	TCCGTCAGCCGTAGTTTTTC	CGTTTGTGCAAATCGAACAT
Imd	FJ546128.1	TGTTAACGACCGATGCAAAA	CATCGCTCTTTTCGGATGTT
Relish	XM_062082689.1	GATGCAGAAGATGAAAAAGCAG	TGAACACATTTCGTTTGTTGTTT
Dicer-like	XM_016917734.1	AGCAGTAGCTGATTGTGTGGA	ATTTCAGAAGCGCAAGGCAT
Ago2	XM_062083470.1	GTGCTCATACATTATCCGTGCAT	TGACCTCGAGTGAACCTCGT
Abaecin	NM_001011617.1	CAGCATTCGCATACGTACCA	GACCAGGAAACGTTGGAAAC
Defensin-1	NM_001011616.2	TGCGCTGCTAACTGTCTCAG	AATGGCACTTAACCGAAACG
ATG7	XM_006564507.2	GGGTTGCACATCCTACTCCC	GCCTGGATTGGAACTTGTGC
TAK1	XM_062075735.1	AAACATCCTGCGTGTCTGGT	TTCCCTCGACGAGAAGCAAC
TAB2	XM_062084844.1	TTGCTTTTCCCTGCATTGCC	ACCAGACACGCAGGATGTTT
Hopscotch	XM_062075185.1	TCAATTACGTTCAGGTACTCTTGT	CAAGATGCCAAAGAGCAGCG
Stat92E	XM_397181.6	TGGAGTCGGTGGACAAACAC	CGTAGCTGGAAGCCACTGAT
Spaetzle	XM_062083110.1	TGCACAAATTGTTTTTCCTGA	GTCGTCCATGAAATCGATCC
Hymenoptaecin	NM_001011615.1	CTCTTCTGTGCCGTTGCATA	GCGTCTCCTGTCATTCCATT
Apidaecin	NM_001011613.1	TTTTGCCTTAGCAATTCTTGTTG	GTAGGTCGAGTAGGCGGATCT
Hsp90	XM_017059135.3	TAGAAATCGGGCGTGCTTCC	GTAGCTTCGCGTCACTCTCA
ATG8	XM_017055098.3	ACGCATGCACTCTCACGTAT	TGTCCTCCCTTTTGCTTTGA
ATG9	XM_017066896.3	TGCACAAACGCGATACATCA	AGGTACCCACCAAATGAACTTGT
ATG10	XM_017053525.3	GATGGATGGGATCTTCGAGGT	CTCGATGGGACAAGTGGCTT

**TABLE 3:** Primer sequences for detecting the virus copies

Gene ID	Forward primer	Reverse primer
CSBV	CCTGGGAAGTTTGCTAGTATTTACG	CCTATCACATCCATCTGGGTCAG
CSBV-Probe	5’-(FAM) CGACATACCCGCAAATTCAGCACGC (Eclipse)-3’	
DWV	CCTGTTCACCGTTTAATAGG	CCAGCACTAGTATTCCAAGA
DWV-Probe	5’-(FAM) AACCAGGCACACCACATACAGCA (Eclipse)-3’	
ABPV	CATATTGGCGAGCCACTATG	CTACCAGGTTCAAAGAAAATTTC
ABPV-Probe	5’-(FAM) ATAGTTAAAACAGCTTTTCACACTG (TRAMA)-3’	

### Application of the online analysis software

#### *AF9* sequence homology analysis

The complete amino acid sequences of bee-derived *AF9* (GenBank: XM_017049815.3) and human-derived *MLLT3* (GenBank: NM_001286691.2) were downloaded from the National Center for Information (NCBI) (https://www.ncbi.nlm.nih.gov). A pairwise sequence alignment was performed using Novopro (https://www.novopro.cn/tools/needle.html), and the homology between the two sequences was determined based on the Needleman-Wunsch algorithm.

#### Protein evolutionary analysis

The protein evolutionary analysis was conducted using the Molecular Evolutionary Genetics Analysis (MEGA, https://www.megasoftware.net/dload_win_gui) for the human-derived MLLT3 and bee-derived AF9.

#### Amino acid functional domain prediction

The functional domain prediction of the amino acids in the bee-derived AF9 was conducted using the Simple Modular Architecture Research Tool (SMART, http://smart.embl-heidelberg.de/), and the Illustrator for Biological Sequences (IBS, http://ibs.biocuckoo.org/online.php) was used to create a predictive diagram of the amino acid functional domains.

#### Protein sequence alignment

The protein sequences of the bee-derived AF9 and human-derived MLLT3 were aligned using ClustalW (http://www.genome.jp/tools-bin/clustalw#clustalw.aln), and Espript 3.0 (http://espript.ibcp.fr/ESPript/ESPript/index.php) was utilized to produce a protein sequence alignment diagram.

#### Genomic m6A modification site prediction

The m6A modification sites of the full gene sequence of *AF9* were predicted using SRAMP (http://www.cuilab.cn/sramp/). Scoring was based on reliability to ultimately identify the most likely gene locations for m6A modification. This software was also used for mapping.

### ELISA quantification of m6A methylation in total RNA

The level of m6A methylation in total RNA was quantified using the m6A RNA Methylation Assay Kit (colorimetric) (EpigenTek, P-9005), as previously described. Briefly, total RNA was extracted from the larval hemolymph using a total RNA extraction kit (Biosharp, Cat. No. BS258A). The total RNA samples were incubated with a binding solution at 37°C for 90 minutes. After measurement, the percentage of m6A in total RNA was calculated as suggested by the manufacturer, wherein m6A% = ([Sample_OD_ – NC_OD_] ÷ *S*) / ([PC_OD_ – NC_OD_] ÷ *P*) × 100%, where NC is the negative control, PC is the positive control, *S* is the amount of the sample RNA, and *P* is the amount of the positive control.

### MeRIP-qPCR

m6A modifications on individual transcripts were determined using a methylated RNA immunoprecipitation (MeRIP) assay with the CUT&RUN m6A RNA Enrichment (MeRIP) Kit (EpigenTek, P9018). Briefly, 20 mg of total RNA from larval hemolymph was immunoprecipitated using antibody-bound magnetic beads, then fragmented to lengths of 70–150 nt. m6A-modified RNAs were then immunoprecipitated and subsequently reverse transcribed for qPCR using primers designed from *AF9*. The level of relative enrichment of m6A was normalized as %Input = 2^–(Ct[IP] – Ct[Input] – log 2[Input dilution factor])^.

### Separation of hemolymph from honeybee larvae

To separate the hemolymph from larvae, sterilized ophthalmic tweezers were used to lift the tail of larva, while another set of tweezers was used to puncture the head of the bee larva, causing the hemolymph to spill from the wound. A 20 μL pipette was used to collect the hemolymph. The hemolymph collected from each larva was placed in a pre-cooled 1.5 mL Eppendorf tube containing 1 μL of anticoagulant.

### Trehalose content and total protein analysis

To evaluate trehalose and protein levels, approximately 5 mg of benzylthiouracil (final concentration of 2.5% wt/vol) was added to a 1.5 mL anticoagulant tube containing the hemolymph to inhibit coagulation. The hemolymph samples from three bee larvae were centrifuged at 12,000 rpm for 10 minutes at 4°C to collect the precipitate. A trehalose content assay kit (Nanjing Jiancheng Bioengineering Institute, A149-1-1) was used to assess trehalose concentrations in the hemolymph. The total protein content in hemolymph was measured using SDS-PAGE and the BSA Protein Assay Kit (Takara Bio; T9300A) according to the manufacturer’s instructions.

### Analysis of trace metal concentrations

To measure copper and zinc concentrations, each group of bee larvae comprising six larvae was used for analysis, with 10 μL of hemolymph taken from each larva placed in a laser confocal culture dish. After adding 200 μL of 4% paraformaldehyde for fixation for 10 minutes, 200 μL of 0.2% Triton X-100 was added and incubated at room temperature for 15 minutes. Then, 200 μL of the Coppersensor 3 (CymitQuimica) and FluoZin-3 indicators (Thermo Fisher Scientific) was added for dark staining for 10 minutes. The samples were rinsed with PBS three times for 5 minutes each time. A DAPI (4’,6-diamidino-2-phenylindole) working solution (DAPI stock solution diluted with PBS at a ratio of 1 [stock solution]:5,000 [PBS]) was used for staining at room temperature for 1–5 minutes. The distributions of copper and zinc ions in the hemolymph of bee larvae were then observed using a laser confocal microscope.

### Cell counting and detection of phenol oxidase activity

To count cells and detect phenol oxidase activity, each group of bee larvae comprised three individuals. Then, 15 μL of hemolymph was collected from each larva and mixed with 0.04% trypan blue staining solution at a 2:1 ratio. Then, 10 μL of the solution was placed onto a hemocytometer to count the abundances of live and dead cells within 3 minutes using an inverted microscope.

To measure phenol oxidase activity, 15 μL of hemolymph was collected from three bee larvae in each group. A phenol oxidase assay kit (Nanjing Jiancheng Bioengineering Institute, H247-1-2) was then used to conduct measurements according to the manufacturer’s instructions.

### Survival statistics

We identified larvae that were soft as mud, did not have the ability to curl up, had inactive mouthparts and head, no longer showed feeding behavior, and were black in color as dead.

We identified pupae with exposed heads and dry epidermis that turned dark brown or black in color as dead.

### Statistical analyses

To mitigate biological bias, larvae were randomly distributed into each group. All data points were biological and not technical replicates. Data are shown as means ± standard deviation (SD), and their significant differences were analyzed and compared in the GraphPad Prism 9 program (MDF Co. Ltd.) using either Student’s *t*-tests (for pairwise comparisons; *P < 0.05, **P < 0.01, ***P < 0.001) or a one-way analysis of variance (ANOVA) test with a post hoc least significant difference test (LSD, for multiple comparisons; *P* < 0.05).

## Data Availability

Raw and processed sequencing data sets analyzed in this study have been deposited to the NCBI Sequence Read Archive (SRA) database under accession number PRJNA1068023.

## SUPPLEMENTAL MATERIAL

Supplemental material jvi.02117-24-s0001.pdf Figures S1 and S2; Tables S1 to S4.

ASM does not own the copyrights to Supplemental Material that may be linked to, or accessed through, an article. The authors have granted ASM a non-exclusive, world-wide license to publish the Supplemental Material files. Please contact the corresponding author directly for reuse.

## References

[1] Sun L, Zhang X, Xu S, Hou C, Xu J, Zhao D, Chen Y. 2021. Antiviral activities of a medicinal plant extract against sacbrood virus in honeybees. Virol J 18:83. doi:10.1186/s12985-021-01550-y 33882983 PMC8059305

[2] Mingxiao M, Ming L, Jian C, Song Y, Shude W, Pengfei L. 2011. Molecular and biological characterization of Chinese sacbrood virus LN isolate. Comp Funct Genomics 2011:409386. doi:10.1155/2011/409386 21527980 PMC3061217

[3] Chen Y, Evans J, Feldlaufer M. 2006. Horizontal and vertical transmission of viruses in the honey bee, Apis mellifera. J Invertebr Pathol 92:152–159. doi:10.1016/j.jip.2006.03.010 16793058

[4] McMenamin AJ, Daughenbaugh KF, Parekh F, Pizzorno MC, Flenniken ML. 2018. Honey bee and bumble bee antiviral defense. Viruses 10:395. doi:10.3390/v10080395 30060518 PMC6115922

[5] Li M, Fei D, Sun L, Ma M. 2019. Genetic and phylogenetic analysis of Chinese sacbrood virus isolates from Apis mellifera. PeerJ 7:e8003. doi:10.7717/peerj.8003 31741790 PMC6858986

[6] Ma M. 2014. New insights of sacbrood virus. Virol Sin 29:410–413. doi:10.1007/s12250-014-3540-9 25511927 PMC8206423

[7] Wang M, Xiao Y, Li Y, Wang X, Qi S, Wang Y, Zhao L, Wang K, Peng W, Luo G-Z, Xue X, Jia G, Wu L. 2021. RNA m6A modification functions in larval development and caste differentiation in honeybee (Apis mellifera). Cell Rep 34:108580. doi:10.1016/j.celrep.2020.108580 33406439

[8] Yongbo Pan YG, Liu T, Zhang Q, Yang F, Duan L, Cheng S, Zhu X, Xi Y, Chang X, Ye Q, Gao S. 2023. Epitranscriptic regulation of HRAS by N6-methyladenosine drives tumor progression. PNAS. doi:10.1073/pnas.230229112 PMC10083612 36996116

[9] Chen YG, Chen R, Ahmad S, Verma R, Kasturi SP, Amaya L, Broughton JP, Kim J, Cadena C, Pulendran B, Hur S, Chang HY. 2019. N6-methyladenosine modification controls circular RNA immunity. Mol Cell 76:96–109. doi:10.1016/j.molcel.2019.07.016 31474572 PMC6778039

[10] Rockwell AL, Hongay CF. 2020. Dm Ime4 depletion affects permeability barrier and Chic function in Drosophila spermatogenesis. Mech Dev 164:103650. doi:10.1016/j.mod.2020.103650 33038528

[11] Wang X, Li Z, Zhang Q, Li B, Lu C, Li W, Cheng T, Xia Q, Zhao P. 2018. DNA methylation on N6-adenine in lepidopteran Bombyx mori. Biochimica et Biophysica Acta (BBA) - Gene Regulatory Mechanisms 1861:815–825. doi:10.1016/j.bbagrm.2018.07.013 30071347

[12] Gokhale NS, McIntyre ABR, McFadden MJ, Roder AE, Kennedy EM, Gandara JA, Hopcraft SE, Quicke KM, Vazquez C, Willer J, Ilkayeva OR, Law BA, Holley CL, Garcia-Blanco MA, Evans MJ, Suthar MS, Bradrick SS, Mason CE, Horner SM. 2016. N6-methyladenosine in flaviviridae viral RNA genomes regulates infection. Cell Host Microbe 20:654–665. doi:10.1016/j.chom.2016.09.015 27773535 PMC5123813

[13] Zhang X, Zhang Y, Dai K, Liang Z, Zhu M, Pan J, Zhang M, Yan B, Zhu H, Zhang Z, Dai Y, Cao M, Gu Y, Xue R, Cao G, Hu X, Gong C. 2020. N6-methyladenosine level in silkworm midgut/ovary cell line is associated with Bombyx mori nucleopolyhedrovirus infection. Front Microbiol 10. doi:10.3389/fmicb.2019.02988 PMC6965365 31998272

[14] Burciaga RA, Ruiz-Guzmán G, Lanz-Mendoza H, Krams I, Contreras-Garduño J. 2023. The honey bees immune memory. Dev Comp Immunol 138:104528. doi:10.1016/j.dci.2022.104528 36067906

[15] Lang S, Simone-Finstrom M, Healy K, Lopez-Uribe M. 2022. Context-dependent viral transgenerational immune priming in honey bees (Hymenoptera: Apidae). J Insect Sci 22:19. doi:10.1093/jisesa/ieac001 PMC8826052 35137131

[16] Guo L, Tang J, Tang M, Luo S, Zhou X. 2023. Reactive oxygen species are regulated by immune deficiency and Toll pathways in determining the host specificity of honeybee gut bacteria. Proc Natl Acad Sci U S A 120:e2219634120. doi:10.1073/pnas.2219634120 37556501 PMC10438842

[17] Parekh F, Daughenbaugh KF, Flenniken ML. 2021. Chemical stimulants and stressors impact the outcome of virus infection and immune gene expression in honey bees (Apis mellifera). Front Immunol 12:747848. doi:10.3389/fimmu.2021.747848 34804032 PMC8596368

[18] Trytek M, Buczek K, Zdybicka-Barabas A, Wojda I, Borsuk G, Cytryńska M, Lipke A, Gryko D. 2022. Effect of amide protoporphyrin derivatives on immune response in Apis mellifera. Sci Rep 12:14406. doi:10.1038/s41598-022-18534-9 36002552 PMC9402574

[19] Özgör E. 2021. The effects of Nosema apis and Nosema ceranae infection on survival and phenoloxidase gene expression in Galleria mellonella (Lepidoptera: Galleriidae) compared to Apis mellifera. Insects 12:953. doi:10.3390/insects12100953 34680722 PMC8538655

[20] Lang H, Wang H, Wang H, Zhong Z, Xie X, Zhang W, Guo J, Meng L, Hu X, Zhang X, Zheng H. 2023. Engineered symbiotic bacteria interfering Nosema redox system inhibit microsporidia parasitism in honeybees. Nat Commun 14:2778. doi:10.1038/s41467-023-38498-2 37210527 PMC10199888

[21] Guo Z, Bai Y, Zhang X, Guo L, Zhu L, Sun D, Sun K, Xu X, Yang X, Xie W, Wang S, Wu Q, Crickmore N, Zhou X, Zhang Y. 2024. RNA m6A methylation suppresses insect juvenile hormone degradation to minimize fitness costs in response to a pathogenic attack . Adv Sci (Weinh) 11. doi:10.1002/advs.202307650 PMC10853702 38087901

[22] Wang P, Doxtader KA, Nam Y. 2016. Structural basis for cooperative function of Mettl3 and Mettl14 methyltransferases. Mol Cell 63:306–317. doi:10.1016/j.molcel.2016.05.041 27373337 PMC4958592

[23] Wang X, Feng J, Xue Y, Guan Z, Zhang D, Liu Z, Gong Z, Wang Q, Huang J, Tang C, Zou T, Yin P. 2016. Structural basis of N(6)-adenosine methylation by the METTL3-METTL14 complex. Nature New Biol 534:575–578. doi:10.1038/nature18298 27281194

[24] Lua J, Liua X, Lie X, Li H, Shia L, Xia X, F.Meyer T, Li X, Sun H, aX Y. 2023. Copper regulates the host innate immune response against bacterial infection via activation of ALPK1 kinase. PNAS. doi:10.1073/pnas.2311630121 PMC10823219 38232278

[25] Kaustav Das Gupta J, Tarique AA, Schembri R, Fantino E, Sly PD, Sweet MJ. 2023. CFTR is required for zinc- mediated antibacterial defense in human macrophages. PNAS. doi:10.1073/pnas.2315190121 PMC10895263 38363865

[26] Ongus JR, Peters D, Bonmatin J-M, Bengsch E, Vlak JM, van Oers MM. 2004. Complete sequence of a picorna-like virus of the genus Iflavirus replicating in the mite Varroa destructor. J Gen Virol 85:3747–3755. doi:10.1099/vir.0.80470-0 15557248

[27] Wei R, Cao L, Feng Y, Chen Y, Chen G, Zheng H. 2022. Sacbrood virus: a growing threat to honeybees and wild pollinators. Viruses 14:1871. doi:10.3390/v14091871 36146677 PMC9505205

[28] Deng Y, Zhao H, Shen S, Yang S, Yang D, Deng S, Hou C. 2020. Identification of immune response to sacbrood virus infection in Apis cerana under natural condition. Front Genet 11:587509. doi:10.3389/fgene.2020.587509 33193724 PMC7649357

[29] Guo Y, Zhang Z, Zhuang M, Wang L, Li K, Yao J, Yang H, Huang J, Hao Y, Ying F, Mannan H, Wu J, Chen Y, Li J. 2021. Transcriptome profiling reveals a novel mechanism of antiviral immunity upon sacbrood virus infection in honey bee larvae (Apis cerana). Front Microbiol 12. doi:10.3389/fmicb.2021.615893 PMC8208235 34149631

[30] Phokasem P, Liuhao W, Panjad P, Yujie T, Li J, Chantawannakul P. 2021. Differential viral distribution patterns in reproductive tissues of Apis mellifera and Apis cerana drones. Front Vet Sci 8. doi:10.3389/fvets.2021.608700 PMC8024463 33842568

[31] Shan L, Liuhao W, Jun G, Yujie T, Yanping C, Jie W, Jilian L. 2017. Chinese sacbrood virus infection in Asian honey bees (Apis cerana cerana) and host immune responses to the virus infection. J Invertebr Pathol 150:63–69. doi:10.1016/j.jip.2017.09.006 28916146

[32] Zhang Y, Li Z, He X, Wang Z, Zeng Z. 2023. H3K4me1 modification functions in caste differentiation in honey bees. IJMS 24:6217. doi:10.3390/ijms24076217 37047189 PMC10094490

[33] Li Y, Zhang L-Z, Yi Y, Hu W-W, Guo Y-H, Zeng Z-J, Huang Z-Y, Wang Z-L. 2017. Genome-wide DNA methylation changes associated with olfactory learning and memory in Apis mellifera. Sci Rep 7. doi:10.1038/s41598-017-17046-1 PMC5717273 29208987

[34] He XJ, Jiang WJ, Zhou M, Barron AB, Zeng ZJ. 2019. A comparison of honeybee (Apis mellifera) queen, worker and drone larvae by RNA-Seq. Insect Sci 26:499–509. doi:10.1111/1744-7917.12557 29110379

[35] Calvanese V, Nguyen AT, Bolan TJ, Vavilina A, Su T, Lee LK, Wang Y, Lay FD, Magnusson M, Crooks GM, Kurdistani SK, Mikkola HKA. 2019. MLLT3 governs human haematopoietic stem-cell self-renewal and engraftment. Nature 576:281–286. doi:10.1038/s41586-019-1790-2 31776511 PMC7278275

[36] Li Y, Wen H, Xi Y, Tanaka K, Wang H, Peng D, Ren Y, Jin Q, Dent SYR, Li W, Li H, Shi X. 2014. AF9 YEATS domain links histone acetylation to DOT1L-mediated H3K79 methylation. Cell 159:558–571. doi:10.1016/j.cell.2014.09.049 25417107 PMC4344132

[37] Yao S, Kang J, Guo G, Yang Z, Huang Y, Lan Y, Zhou T, Wang L, Wei C, Xu Z, Li Y. 2022. The key micronutrient copper orchestrates broad-spectrum virus resistance in rice. Sci Adv 8. doi:10.1126/sciadv.abm0660 PMC10883364 35776788

[38] Chen B, Yu P, Chan WN, Xie F, Zhang Y, Liang L, Leung KT, Lo KW, Yu J, Tse GMK, Kang W, To KF. 2024. Cellular zinc metabolism and zinc signaling: from biological functions to diseases and therapeutic targets. Signal Transduct Target Ther 9:6. doi:10.1038/s41392-023-01679-y 38169461 PMC10761908

[39] Maryann Morales MYY, Goddard WA, Gray HB, Winkler JR. 2024. Copper(II) coordination to the intrinsically disordered region of SARS- CoV- 2 Nsp1. PNAS. doi:10.1073/pnas.2402653121 PMC11098128 38722808

[40] Guo X, Mutch M, Torres AY, Nano M, Rauth N, Harwood J, McDonald D, Chen Z, Montell C, Dai W, Montell DJ. 2024. The Zn^2+^ transporter ZIP7 enhances endoplasmic-reticulum-associated protein degradation and prevents neurodegeneration in Drosophila. Dev Cell 59:1655–1667. doi:10.1016/j.devcel.2024.04.003 38670102 PMC11233247

[41] Murata D, Roy S, Lutsenko S, Iijima M, Sesaki H. 2024. Slc25a3-dependent copper transport controls flickering-induced Opa1 processing for mitochondrial safeguard. Dev Cell 59:2578–2592. doi:10.1016/j.devcel.2024.06.008 38986607 PMC11461135

[42] Traynor KS, Mondet F, de Miranda JR, Techer M, Kowallik V, Oddie MAY, Chantawannakul P, McAfee A. 2020. Varroa destructor: a complex parasite, crippling honey bees worldwide. Trends Parasitol 36:592–606. doi:10.1016/j.pt.2020.04.004 32456963

