# Supplementary material for: Chinese sacbrood virus mediates m6A modification to target and suppress the expression of hemolymph maintenance gene AF9, exacerbating bee infections
Source: J Virol. 2025 Feb 3;99(3):e02117-24. doi: 10.1128/jvi.02117-24 (PMC11915840; doi:10.1128/jvi.02117-24)
Supplement: Supplemental material — Figures S1 and S2; Tables S1 to S4. [file jvi.02117-24-s0001.pdf]

Fig.S1

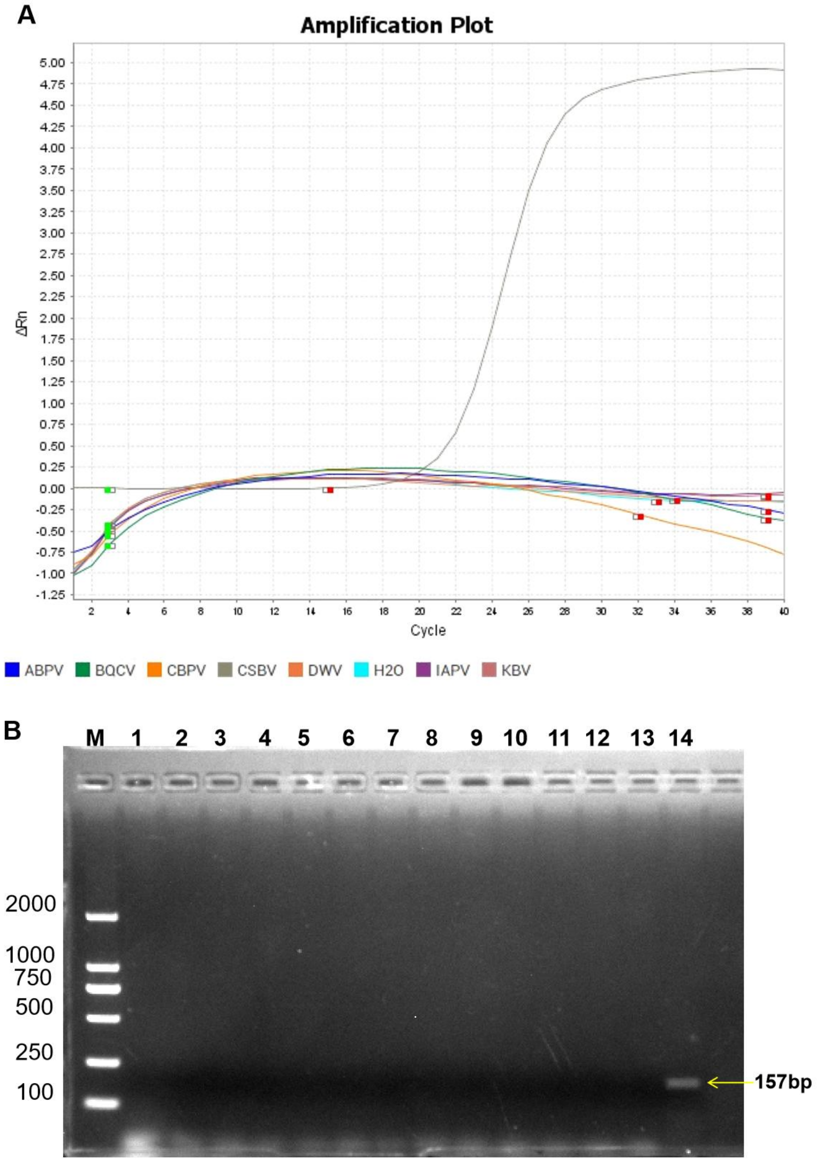

M: 2000 marker; 1:Negative control; 2:ABPV; 3:BQPV; 4:CBPV; 5:DWV-A; 6:DWV-B; 7:DWV-C; 8:IAPV; 9:KBV; 10:KV; 11:AmFV; 12:LSV; 13:VDV; 14: CSBV

Fig.S1 The virus purification solution was identified by RT-PCR(A) and PCR(B).

Fig.S2

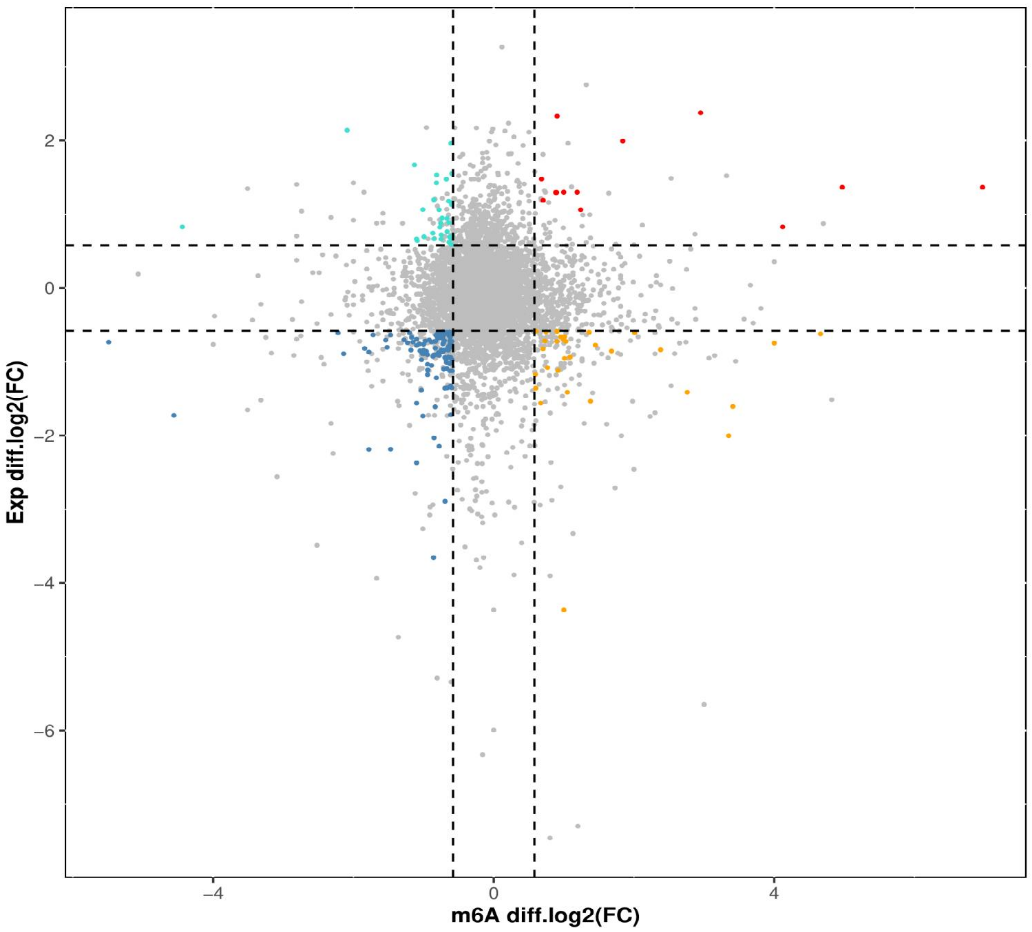

Fig.S2 Combined analysis of m6A abundance and mRNA expression after CSBV infection (Four-quadrant plot showing the number of peaks contained in differentially expressed mRNAs).

Table S1.Primer sequence of qPCR

Bee Virus	Forward primer	Reverse primer
DWV	TGTGGTGTAGTAAGCGTCGT	TCATCCGTAGAAAGCCGAGT
BQCV	GCTGCTGCTGCTGCTGCTG	CACACACACACACACACAC
CBPV	AAGCGGAGAGAGAGAGAGA	TCTCTCTCTCTCTCTCTCG
ABPV	ATGTCGTCCTCGTCTTCCTC	TCATCTCCAGCACGTTGTCA
CSBV	GCGGATCCATGGATAAACCGAAGG ATATAAG	GCAAGCTTTTATTGTACGCGCGGT AAATA
KBV	AGCGGAGAGAGAGAGAGAG	TCTCTCTCTCTCTCTCTCC
IAPV	ATGTCGTCCTCGTCTTCCTT	TCATCTCCAGCACGTTGTCT

Table S2.Primer sequence of PCR

Bee Virus	Forward primer	Reverse primer
BQCV	TGGTCAGCTCCCACTACCTTAAAC	GCAACAAGAAGAAACGTAAACCAC
DWV-1	TTCCAGAAGCTCCCAATGCT	GCAATAGGAATATCATTCTC
ABPV	TTATGTGTCCAGAGACTGTAT	GCTCCTATTGCTCGGTTTTTC
KBV	GATGAACGTCGACCTATTGA	TGTGGGTTGGCTATGAGTCA
CSBV	ACCTTCATCCAGTATCAGAACCAT	ATAACCACCCGTCCCAGAG
CBPV	AGTTGTCATGGTTAACAGGATACG AG	TCTAATCTTAGCACGAAAGCCGAG
LSV	CGTGCGGACCTCATTTCTTCATGT	CTGCGAAGCACTAAAGCGTT
IAPV	CCATGCCTGGCGATTCAC	CTGAATAATACTGTGCGTATC
AmFV-1	CCGCAGGCTTCAACGAATTA	GTCTCGGGTAACCACGTACT
KV	AAGACTGATCTTATGGAAAT	TCATTCTCACAATGCTTGCA
VDV	GCCCTGTTCAAGAACATG	CTTTTCTAATTCAACTTCACC
DWV-2	AGGAGGCCAGTGCCTGGTA	TCTTTCAAAATTTTTGGACT
DWV-3	CTTCAAGGAGTATATACTTA	AAACTAATTTTTCTTTCAAA

Table S3. Analysis of mRNA differential expression between groups

Gene ID	Control	Infection	log2FoldChange	pvalue
gene-LOC108000203	3222.94	66446.38	-4.365866858	1.67E-09
gene-LOC107994320	1272.6	2272.62	-0.837517924	8.44E-09
gene-LOC107994987	681.17	1119.55	-0.716941927	2.44E-06
gene-LOC107995138	517.88	800.43	-0.622637729	0.003737216
gene-LOC107995652	218.95	357.98	-0.70083085	0.00122968
gene-LOC107999155	1885.23	3341.12	-0.825934822	3.87E-07
gene-LOC108002262	673.73	1990.61	-1.561219861	1.83E-05
gene-LOC108002480	535.46	848.5	-0.662866338	8.30E-07
gene-LOC108002611	73.58	134.22	-0.854793815	0.029615396
gene-LOC108003197	8560.08	13052.08	-0.608261017	2.73E-05
gene-LOC108003260	191.72	323.92	-0.747290383	0.002033096
gene-LOC108003262	123.98	212.51	-0.774902827	0.006447447
gene-LOC108003854	2772.53	4188.4	-0.594220423	3.65E-05
gene-LOC107992457	4938.62	3051.02	0.695032854	0.007685661
gene-LOC107992733	983.78	619.3	0.667006709	0.000171857
gene-LOC107992772	869.69	451.75	0.943485918	0.009171233
gene-LOC107992887	15255.21	9777.7	0.641680119	1.43E-09
gene-LOC107993415	893.28	428.13	1.061528374	0.004904675
gene-LOC107993784	16842.84	7686.4	1.131760361	0.000304099
gene-LOC107993930	5835.27	3884.34	0.587370089	0.020451086
gene-LOC107995333	1341.8	830.16	0.69191852	0.000508409
gene-LOC107996246	25301.91	14248.61	0.828468455	0.028452508
gene-LOC107996486	708.35	263.5	1.425537504	5.51E-05
gene-LOC107998788	877.57	515.91	0.764952566	2.66E-06
gene-LOC108000235	927.05	554.14	0.744253849	1.27E-06
gene-LOC108003337	797.24	356.15	1.159356641	3.66E-05

Table S4. Fold changes in m6A modification levels between groups

Gene ID	m_FC	Gene ID	m_FC
LOC108000203	1	LOC107992457	-0.994
LOC107994320	2.38	LOC107992733	-0.852
LOC107994987	0.729	LOC107992772	-0.664
LOC107995138	4.66	LOC107992887	-1.09
LOC107995652	0.992	LOC107993415	-0.778
LOC107999155	0.703	LOC107993784	-0.599
LOC108002262	0.67	LOC107993930	-0.602
LOC108002480	0.956	LOC107995333	-0.627
LOC108002611	1.68	LOC107996246	-4.44
LOC108003197	2.01	LOC107996486	-0.817
LOC108003260	4	LOC107998788	-0.664
LOC108003262	1.45	LOC108000235	-0.868
LOC108003854	0.746	LOC108003337	-0.605
